# Monitoring of Nitrification in Chloraminated Drinking Water Distribution Systems With Microbiome Bioindicators Using Supervised Machine Learning

**DOI:** 10.3389/fmicb.2020.571009

**Published:** 2020-09-16

**Authors:** Vicente Gomez-Alvarez, Randy P. Revetta

**Affiliations:** Office of Research and Development, United States Environmental Protection Agency, Cincinnati, OH, United States

**Keywords:** nitrification, bioindicators, receiver-operating characteristic, microbiome, machine learning

## Abstract

Many drinking water utilities in the United States using chloramine as disinfectant treatment in their drinking water distribution systems (DWDS) have experienced nitrification episodes, which detrimentally impact the water quality. Identification of potential predictors of nitrification in DWDS may be used to optimize current nitrification monitoring plans and ultimately helps to safeguard drinking water and public health. In this study, we explored the water microbiome from a chloraminated DWDS simulator operated through successive operational schemes of stable and nitrification events and utilized the 16S rRNA gene dataset to generate high-resolution taxonomic profiles for bioindicator discovery. Analysis of the microbiome revealed both an enrichment and depletion of various bacterial populations associated with nitrification. A supervised machine learning approach (naïve Bayes classifier) trained with bioindicator profiles (membership and structure) were used to classify water samples. Performance of each model was examined using the area under the curve (AUC) from the receiver-operating characteristic (ROC) and precision-recall (PR) curves. The ROC- and PR-AUC gradually increased to 0.778 and 0.775 when genus-level membership (i.e., presence and absence) was used in the model and increased significantly using structure (i.e., distribution) dataset (AUCs = 1.000, *p* < 0.01). Community structure significantly improved the predictive ability of the model beyond that of membership only regardless of the type of data (sequence- or taxonomy-based model) we used to represent the microbiome. In comparison, an ATP-based model (bulk biomass) generated a lower AUCs of 0.477 and 0.553 (ROC and PR, respectively), which is equivalent to a random classification. A combination of eight bioindicators was able to correctly classify 85% of instances (nitrification or stable events) with an AUC of 0.825 (sensitivity: 0.729, specificity: 0.894) on a full-scale DWDS test set. Abiotic-based model using total Chlorine/NH_2_Cl and NH_3_ generated AUCs of 0.740 and 0.861 (ROC and PR, respectively), corresponding to a sensitivity of 0.250 and a specificity of 0.957. The AUCs increased to > 0.946 with the addition of NO_2_^–^ concentration, which is indicative of nitrification in the DWDS. This research provides evidence of the feasibility of using bioindicators to predict operational failures in the system (e.g., nitrification).

## Introduction

Many United States water treatment facilities use chloramine as disinfectant treatment to ensure regulatory compliance of targeted disinfectant by-products (DBPs). However, more than half of water utilities using chloramine as a disinfectant experience episodes of nitrification in their drinking water distribution system (DWDS; Zhang et al., 2009) with detrimental consequences in water quality (e.g., taste, odor; Carrico et al., 2008). Nitrification is a microbial process by which free-ammonia (NH_3_) provides the substrate (from excess or decay of chloramine) for ammonia-oxidizing bacteria (AOB) producing nitrite (NO_2_^–^), which can be oxidized to nitrate (NO_3_^–^) by nitrite-oxidizing bacteria (NOB; Wahman et al., 2013). Changes in water quality have been shown to promote the growth of nitrifying bacteria (i.e., nitrification). Background nitrification (complete or partial) likely occurs in small localized areas in all chloraminated systems (Carrico et al., 2008), but adequate levels of disinfectant residual (i.e., chloramines) appear to prevent the intensity and propagation of nitrification in DWDS (Wahman et al., 2013). However, once nitrification occurs, chloramine is rapidly degraded and the current approach to stop and control nitrification is the periodic switching of the disinfectant from chloramine to free chlorine (i.e., a ‘chlorine burn’; Carrico et al., 2008).

Many United States water facilities have adopted water quality monitoring plans to assess DWDS water quality and determine when nitrification is occurring (Zhang et al., 2009; Hill and Arweiler, 2013). These plans typically incorporate the physico-chemical monitoring of some or all of the following water quality parameters (i.e., water indicators of nitrification): (i) low or depleted total chlorine residual (chloramine degradation), (ii) elevated NO_2_^–^ concentration, (iii) elevated or depleted free NH_3_ concentration (nitrification occurring, e.g., source of nutrient), (iv) increased NO_3_^–^ concentration, (v) low pH, and (vi) increased temperature (> 15°C as an indicator of nitrification potential, e.g., summer). Among the monitoring strategies, the biomonitoring approach has become a popular alternative to observe environmental changes and analyze signals for possible ecological shifts (Siddig et al., 2016). Importantly, biomonitoring may reveal the potential onset of a nitrification episode before sufficient levels of chemical surrogates are detected as changes in the biological stability of the ecosystem may represent early events in the pathway leading to failed systems. The term biomonitoring has been defined as the use of biological communities or their responses as an indicator of the quality of the system and are used mostly to monitor chemical changes in the environment (McCarty et al., 2002; Bartell, 2006). For example, biomonitoring determined by culture-based heterotrophic plate count (HPC) and AOB most probable number (MPN) counts in combination with chemical parameters are commonly assessed to identify events of nitrification in DWDS (Regan et al., 2002; Hoefel et al., 2005). Although it has been reported that increased HPC counts correlate with episodes of nitrification (Skadsen, 1993), this standard culture-based method is not considered a useful operational indicator because (i) the incubation time (> 72 h and up to a month for HPC and MPN, respectively) and (ii) elevated HPC counts can occur for a number of reasons (e.g., contamination in the DWDS, seasonal changes). Culture-independent techniques have created new opportunities for assessment of DWDS operations (Zhang et al., 2009) and current advances in sequencing technology (e.g., high-throughput and deep DNA sequencing) allow for the direct assessment and diversity of the water microbiome present in DWDS. This technology is being increasingly utilized to reveal spatial and temporal dynamics of microbial communities in DWDS (Shaw et al., 2015; Bautista-de los Santos et al., 2016; Gomez-Alvarez et al., 2016).

**FIGURE 1:**
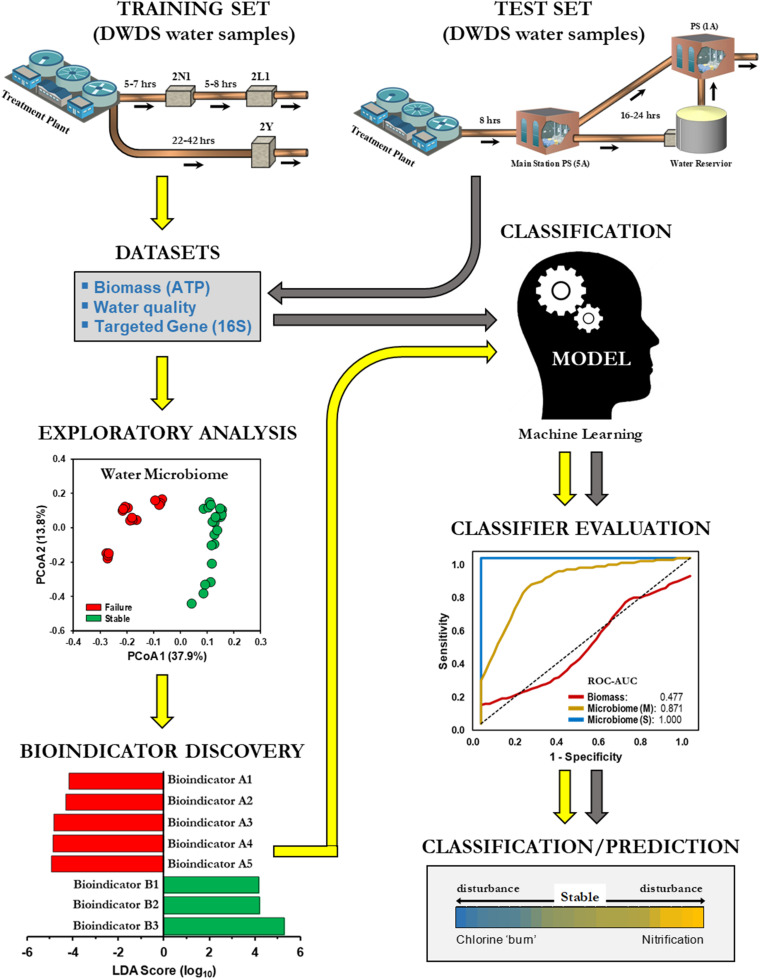
Machine learning classification for the prediction of drinking water distribution systems (DWDS). Schematic of a supervised machine learning approach to classify operational schemes. Different color arrows indicate training (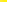) and test (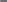) datasets.

This study investigated the practicality of bulk water (BW) microbiome-based signatures as a screening tool and a potential predictor of nitrification in DWDS. BW was chosen because sampling of BW in a DWDS by water utility operators is relatively simpler and easier than collecting biofilm samples from underground pipes. For this purpose, we initially examined the BW bacterial community of a chloraminated DWDS simulator operated through successive operational schemes, including an episode of nitrification (Gomez-Alvarez et al., 2016). DWDS simulators provide a managed platform to review all related water indicators (biotic and abiotic) along with an assessment of system operations (i.e., schemes), since several related events can occur simultaneously or sequentially (Hill and Arweiler, 2013). Our methodology applied a supervised classification machine learning approach (naïve Bayes algorithm) for developing predictive models for drinking water instability (e.g., nitrification; Figure 1). Classification models were trained with indicator species profiles (hereinafter referred to as *bioindicators*) generated from high-throughput 16S rRNA gene libraries. The training sets were divided into two groups (i.e., binary) of positives and negatives (Failure and Stable, respectively). The objective in binary classification is to train (learn) a model (function) that can distinguish one operational scheme from another. After method evaluation, our models were compared against current monitoring indicators of nitrification [biomass (ATP) and water chemical parameters]. In addition, we utilized our microbiome-based classification model to determine the operational scheme of full-scale DWDS studies of chloraminated systems. Nitrification can lead to the degradation of water quality in DWDS and can potentially impact compliance with the Safe Drinking Water Act (Friedman et al., 2013). The results of this study demonstrated the feasibility of using BW microbiome-based screening tools to assess water quality, thus expanding the inventory of molecular tools for the prediction of nitrification in DWDS.

## Materials and Methods

### Water Microbiome and Data Source

**FIGURE 2:**
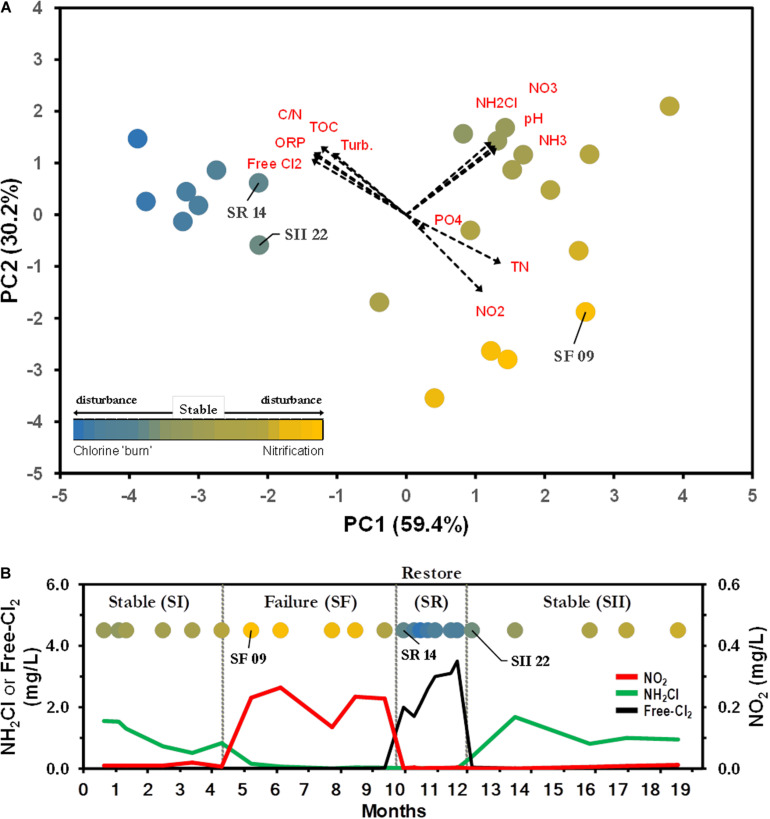
Water parameters distinguish between operational schemes. **(A)** Principal component (PC) analysis representing the relationship of drinking water distribution systems (DWDS) simulator samples. Values in parenthesis indicate the percentage of total variation explained by the first two axes. Dashed arrows indicate the orientation and contribution of water parameters to the ordination plot. Labeled samples represent transition points. Bulk water (BW) source: SS (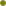), SF (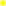), SR (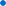). Water quality values are listed in Supplementary Table S1. **(B)** The simulator was operated through four successive operational schemes; a stable period (SI) where chloramine residual (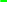) is maintained to a failure period (SF) where no chloramine residual is maintained as a result of nitrification (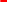), followed by a ‘chlorine burn’ (SR) by switching disinfectant from chloramine to free chlorine (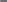) and switching back to chloramine resuming normal operation (SII).

To investigate the BW bacterial microbiome composition within and between different operational schemes, we re-examined the dataset compiled by Gomez-Alvarez et al. (2016). The study examined the microbial communities of a chloraminated DWDS simulator operated through successive operational schemes, including an episode of nitrification, followed by a ‘chlorine burn’ [restore event (SR)] by switching disinfectant from chloramine to free chlorine (Figure 2). DWDS simulator design, water analysis, sample collection, DNA extraction, sequencing, and biomass quantification were previously published (Gomez-Alvarez et al., 2016) and briefly described in Supplementary Material.

The current study re-examined the 16S rRNA gene (v4 region) sequence dataset generated using Illumina MiSeq sequencing technology and only BW sequences associated with Stable (SS; SI and SII) and Failure (SF) events. In addition, water quality parameters (Supplementary Table S1) and biomass values corresponding to the same sampling points were included in this study.

### Operational Schemes

Stable (SS) and Failure (SF) operational schemes were defined using the water quality indicators recommended by the American Water Works Association (Hill and Arweiler, 2013). Low total chlorine residual (< 0.2 mg/L) and increased concentrations of NO_2_^–^ (> 0.05 mg/L) and free NH_3_ (> 0.2 mg/L) are indicative of nitrification (i.e., Failure). Stable systems maintain a baseline of > 1.5 mg/L total chlorine residual, < 0.15 mg/L free NH_3_, and < 0.01 mg/L NO_2_^–^.

### Water Chemical Parameters

Eleven water chemical parameters [pH, turbidity, NH_2_Cl, Free-Chlorine, NH_3_, NO_2_^–^, NO_3_^–^, phosphate (P), total organic carbon (TOC), total nitrogen (TN), and C/N ratio] were used as variable inputs for principal component analysis (PCA). Prior to the analysis, water parameters were log-transformed to decrease the variability of data due to differences in magnitudes and scales of measurements (Kenkel, 2006). A non-parametric Mann–Whitney *U* tests (α = 0.05) was used to determine the differences in the water quality parameters between SS and SF schemes. PCA ordination and Wilcoxon–Mann–Whitney *U* tests were performed using the software PAST v3.12 (Hammer et al., 2001).

### 16S rRNA Sequence Analysis

Reads were analyzed using the software MOTHUR v1.37.6 (Schloss et al., 2009) and were screened following the procedure described in Gomez-Alvarez et al. (2016) and briefly described in Supplementary Material. Reads were aligned against the SILVA SEED release 123 reference dataset and grouped with 97% sequence identity as the cut-off point for each operational taxonomic unit (OTU). Taxonomic classification was obtained using the Ribosomal Database Project (RDP v16) reference database. The sequences and taxonomic outlines for the RDP hierarchies were downloaded from the MOTHUR website^1^. Prior to community analysis, samples were rarefied to the smallest dataset (5,000 reads).

### Microbial Community Assemblages

Normalized libraries were used to calculate richness (*S*), richness estimators (ChaoI and *S*_*ACE*_), Shannon diversity (*H*), and evenness (*H*_*E*_) with the software MOTHUR v1.37.6 (Schloss et al., 2009). Principal coordinate analysis (PCoA) based on the Square Root Jensen–Shannon Divergence coefficient (dissimilarity) was used to describe the relationships among microbial communities. The Jensen–Shannon divergence is a method of measuring the similarity between two probability distributions based on relative distribution. A Mann–Whitney *U* test (α = 0.05) was used to test the differences between the diversity indices of samples from the SS and SF schemes.

One-way PERMANOVA test (Anderson, 2001) based on Jensen–Shannon dissimilarity matrix derived from the distribution of microbial communities with 9,999 permutations was used to determine if there were significant differences (α = 0.05) between the communities at each operational scheme. Similarity Percentage (SIMPER) analysis was used to determine the percentage contribution of species to the differences observed between operational schemes (Clarke, 1993). Ordination plot, Wilcoxon–Mann–Whitney *U* tests, SIMPER, and PERMANOVA were performed with the software PAST v3.12 (Hammer et al., 2001).

### Bioindicator Discovery Analysis

Sequence- and taxonomy-based relative abundance datasets were examined for bioindicator discovery. Sequence-based dataset are reads grouped with 97% sequence identity as the cut-off point (i.e., OTU). Taxonomy-based dataset are reads assigned to the same taxonomy classification (i.e., genus-level bioindicators). Differentially abundant microbial bioindicators were identified using the linear discriminative analysis (LDA) effect size (LEfSe) program v1.0 (Segata et al., 2011) on a Galaxy server hosted by the Huttenhower Lab at Harvard^2^. The parameters used were an LDA threshold score = 2.0 and α = 0.05. LEfSe determines the OTUs or taxa (i.e., features) most likely to explain differences between operational schemes (i.e., classes) by coupling standard tests for statistical significance with biological consistency and effect relevance (Segata et al., 2011).

### Machine Learning Classification and Evaluation

A supervised machine learning approach was used to classify samples from SS and SF (Figure 1). For our experimental approach we used the naïve Bayes classification algorithm, a probability-based method that assumes a strong conditional independence of features (Lowd and Domingos, 2005; Knights et al., 2011). All models were evaluated using the 10-fold cross-validation (Refaeilzadeh et al., 2009). In summary, the training dataset is first randomly divided into ten equal sized segments or folds, the models are trained on nine segments and tested on the tenth segment. The process is repeated ten times using a different segment for testing and then combined to produce a single evaluation score.

Performance of each classification model was displayed by a confusion matrix and evaluated by calculating the area under the curve (AUC) from receiver-operating characteristic (ROC) and precision-recall (PR) curves (Fawcett, 2006; Lever et al., 2016). The confusion matrix indicates the number of instances with regards to the predicted and the actual classes. The AUC, a metric of classification accuracy, was computed on ten sets of predictions obtained from 10-fold cross-validation. An AUC of 1.0 indicates an excellent classifier (no false positive or false negative), while a value of < 0.5 is equivalent to a random classification of the subjects (Fawcett, 2006). A ROC curve represents relative tradeoffs between benefits (TP; true positives) and costs (FP; false positives), in which TP rate is plotted on the *y*-axis and FP rate is plotted on the *x*-axis. PR curve is the TP rate (i.e., recall) against the positive predictive value (PPV) or precision. ROC is routinely used to evaluate classifier performance; however, PR has proven particularly useful in this study where the overall number of positives examples is small, i.e., imbalanced datasets (Brodersen et al., 2010; Saito and Rehmsmeier, 2015). Construction of models and evaluation were conducted using the Knowledge Flow Interface (Supplementary Figure S1) implemented in the Waikato Environment for Knowledge Analysis (WEKA) suite v3.9.0 (Hall et al., 2009).

### Training Sets

Classification models were trained with chosen biotic features of either biomass (ATP), OTUs (Supplementary Figure S2), or genus-level taxonomic groups (Supplementary Table S2) and based on data generated from the DWDS simulator (Gomez-Alvarez et al., 2016). Binary training sets were divided into two groups of positives and negatives (Supplementary Table S3). The objective in binary classification is to train (learn) a model (function) that can distinguish one operational scheme from another (e.g., Failure vs. Stable). To avoid overfitting of the biotic models based on the microbiome, we adopted stringent conditions (LDA = 4.0, *p* < 0.01) in the selection of OTUs and genus-level taxonomic bioindicators (Schubert et al., 2014; Zackular et al., 2014). Training datasets were class-imbalanced with a higher number of “negative” samples (class: Stable) than “positive” samples (class: Failure). To make the model optimize its performance on both the classes, a synthetic minority over-sampling technique (SMOTE) was applied to the imbalanced datasets. SMOTE algorithm generates a random set of artificial data from minority samples (Chawla et al., 2002).

### Test Sets

The test sets included only genus-level taxonomic groups identified as bioindicators (see previous section) compiled from water samples of full-scale DWDS studies of chloraminated systems (Supplementary Tables S4, S5). Species profiles were obtained from published results and/or by reanalyzing their corresponding sequencing data (if available) following the protocol listed in this study (see section “16S rRNA Sequence Analysis”). The following samples were use in the analysis: (i) forty-six locations (sequence libraries: *n* = 137) along the DWDS located in Pinellas County, FL (Wang et al., 2014), (ii) multiple locations (*n* = 13) along two distinct chloramine-treated DWDS in Western Australia (Shaw et al., 2015), (iii) tap water samples (*n* = 21) from five DWDSs along the Arkansas and lower Mississippi Rivers (Holinger et al., 2014), (iv) tap water samples (*n* = 9) from two municipalities located within the headwaters of the Ohio River Basin (Stanish et al., 2016), (v) nine locations (*n* = 116) in the DWDS of Ann Arbor, MI (Pinto et al., 2014), (vi) water samples (*n* = 30) from a building plumbing rig connected to DWDS in the eastern portion of the continental United States (Ji et al., 2015), (vii) tap water samples (*n* = 40) collected in 2012 from two municipal drinking water systems (Algiers and Carrolton) in New Orleans, LA (Hull et al., 2017), and (viii) five locations (*n* = 14) along the DWDS in Urbana, IL (Hwang et al., 2012). Furthermore, eleven water samples from the DWDS simulator obtained before the start of the current experiment were included in the test set.

### Classification Performance and Prediction

Examination of the classification performance our model was performed with a microbiome-based test set of full-scale DWDS *categorized* instances. Categorized instances (*n* = 161) are samples defined as Stable (SS) or Failure (SF) by the authors or using the water quality indicators recommended by the American Water Works Association (Hill and Arweiler, 2013). The test set of *categorized* instances contained 113 SS and 48 SF samples. Conversely, most of the published research studies used in this study omitted the operational status of the DWDS or did not include key water chemical parameters of nitrification (e.g., NO_2_^–^). The latter dataset was defined as *uncategorized* instances (*n* = 230) and was used only to predict the operational scheme of these samples.

Taxonomic identification of 16S rRNA gene reads is usually based on one of these three reference databases: RDP, SILVA or Greengenes (Balvočiūtė and Huson, 2017). Given the known discrepancies of microbial classifications, the choice of a reference database to assign taxonomic affiliation (i.e., features) may affect the ability of our model to classify BW samples. To address this, we constructed three test sets of *categorized* instances with the same bioindicator profile using either the RDP, SILVA or Greengenes as reference database for taxonomic identification. The sequences and taxonomic outlines for the nr SILVA (release 128) and Greengenes (release gg_13_8_99) hierarchies were downloaded from the MOTHUR website (see footnote 1).

In addition, we constructed an abiotic-based model using water chemical parameters [disinfectant (total Chlorine/NH_2_Cl), NH_3_ and NO_2_^–^] to compare the classification performance and prediction against the microbiome-based model. The performance of the classification models was evaluated by plotting the ROC and PR curves using the WEKA suite v3.9.0 (Hall et al., 2009).

## Results

### Water Quality and Operational Schemes

Principal component analysis of water samples based on eleven physico-chemical parameters showed differences between operational schemes (Figure 2A). The first two axes explained 89.6% of the total variation, with chloramine (NH_2_Cl), NH_3_, NO_2_^–^ and free-chlorine as the major contributors of dissimilarity (Supplementary Table S1). The water samples were clustered in three distinctive schemes; Stable (SS), Failure (SF), and Restore (SR). The clustering of samples in the plot was further confirmed by one-way ANOSIM (Global *R* = 0.873, *p* < 0.001; Gomez-Alvarez et al., 2016). The current approach to stop nitrification is the switching of the disinfectant from chloramine to a high concentration of free chlorine (e.g., SR, Supplementary Table S1 and Figure 2B). SR was not included in further analysis, and data from both pre- (SI) and post-Stable (SII) were combined into one SS dataset, as no significant differences were observed between latter events (Gomez-Alvarez et al., 2016). Significant differences in the levels of NH_2_Cl, NH_3_, and NO_2_^–^ were observed between SS and SF water samples (Mann–Whitney *U* tests, *p* < 0.001). SS samples were characterized with a background level of NH_2_Cl (1.03 ± 0.43 Cl_2_ mg L^–1^) and NH_3_ (0.20 ± 0.08 mg L^–1^) with a minimal presence of NO_2_^–^ (Supplementary Table S1 and Figure 2B). SF water samples showed depleted levels of NH_2_Cl and NH_3_, but a higher concentration of NO_2_^–^ (Supplementary Table S1 and Figure 2B).

### Bacterial Richness and Diversity

Forty 16S rRNA BW libraries (Gomez-Alvarez et al., 2016) from 11 sample times were analyzed for this study. A total of 1,090 bacterial OTUs were identified at a ≥ 97% sequence identity cutoff (rarefied to 5,000 reads per library). Only 259 OTUs (24% of the total OTU diversity) were shared by SS and SF, representing 98.7–99.3% of the reads in their respective schemes. Further analysis revealed 546 (32% of the OTUs and 1.8% of reads) and 285 (52% and 0.7%) OTUs found exclusively in the SS and SF libraries, respectively. Taxonomic classification revealed that the majority of the diversity were associated with the phylum *Proteobacteria* (59% of reads), *Actinobacteria* (30%), and *Bacteroidetes* (5%) with additional representatives of 17 phylum detected to a lesser extent (≤ 2% each). Alpha diversity indexes and rarefaction analysis revealed the diversity of bacterial groups contained in this system (Table 1 and Supplementary Figure S3). Observed OTUs and diversity metrics (e.g., *S*_*ACE*_, ChaoI) were slightly higher in the SF compared to the SS communities but did not reach statistical significance (Mann–Whitney *U* tests, *p* > 0.01; Table 1). A more comprehensive and detailed community analysis of the microbial diversity and structure can be found in Gomez-Alvarez et al. (2016).

**TABLE 1:** Alpha diversity indexes (± SD) for microbial bulk water (BW) communities along operational schemes in the pipe-loop system.

Characteristics^§^	Stable^‡^	Failure	*P* value^†^
	SS (*n* = 24)	SF (*n* = 16)	
Observed species (*S*)	121 ± 38	119 ± 12	0.3476
**Richness estimator**			
ChaoI	192 ± 50	198 ± 20	0.6990
*S*_*ACE*_	227 ± 60	246 ± 40	0.1896
Shannon diversity (*H*)	2.11 ± 0.66	2.50 ± 0.14	0.0346
Shannon evenness (*H*_*E*_)	0.44 ± 0.13	0.52 ± 0.02	0.0132

*^§^For comparison, libraries were normalized to 5,000 reads. ^‡^Operational schemes: SS, stable chloramine residual; SF, complete nitrification and minimal chloramine residual. ^†^Significance set at α = 0.01.*

### Microbial Assemblages and Bioindicator Discovery

**FIGURE 3:**
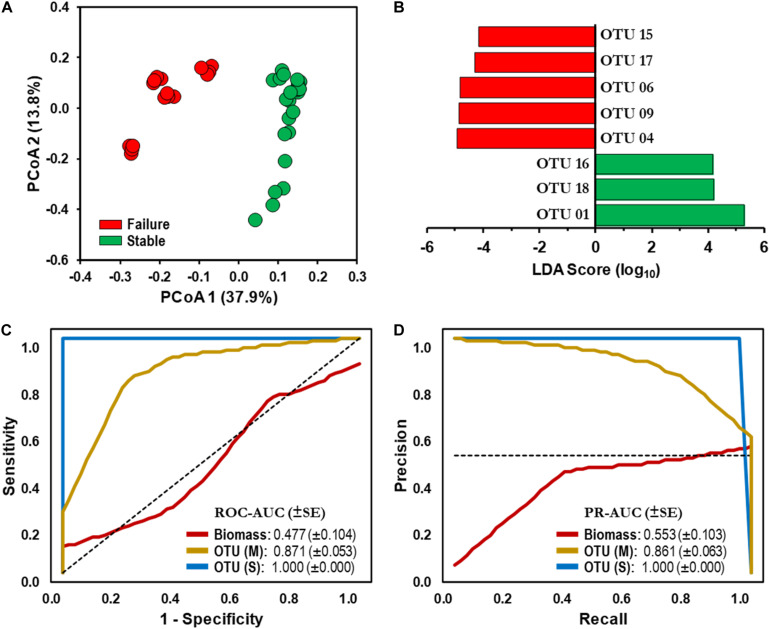
Microbial assemblages and bioindicator discovery. **(A)** Principal coordinate analysis (PCoA) ordination plot based on Jensen–Shannon dissimilarity of 16S rRNA operational taxonomic unit (OTU)-level bacterial profiles (cutoff = 0.03). Values in parenthesis indicate the percentage of total variation explained by the first two axes. Samples: Stable (SS, **
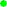
**), Failure (SF, 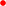). **(B)** Identification of statistically significant genus-level assigned OTU bioindicators using linear discriminative analysis (LDA) effect size (LEfSe) analyses (LDA score > 4.0, *p* < 0.0001). Negative LDA scores are enriched in SF while positive LDA scores are enriched in SS events. **(C)** Receiver operating characteristic (ROC) and **(D)** Precision-recall (PR) curves with area under the curve (AUC) values and 95% confidence intervals in parenthesis for predictive model comparing biomass (ATP, 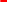) and microbial bioindicators based on community membership [OTU (M), 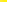] and structure OTU (S), 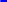] data. Dashed lines indicate the null model. Samples: biomass, *n* = 32; OTU, *n* = 48.

The resulting PCoA ordination plot (*n* = 40) highlighted an evident difference in the community structure between SF and SS schemes (Figure 3A). The separation of samples by operational schemes was confirmed by one-way PERMANOVA test based on Jensen–Shannon dissimilarity matrix (Pseudo-*F* value = 17.47, *p* < 0.0001; Table 2). The microbial communities examined in this study contained ≈49% singletons (i.e., OTU with only one sequence for all samples combined). These OTUs represent the rare biosphere and explained < 0.33% SIMPER analysis of the dissimilarity within the operational schemes. Most of the dissimilarity (≈80%, SIMPER analysis) is explained by a small number of OTUs (22 out of 1,090) whose relative abundance varied significantly among operational schemes. For example, OTU 01 (taxonomic affiliation: *Actinobacteria*) is the dominant representative in the SS with a relative distribution of 43% but only 8% in the SF scheme (Supplementary Figure S2). While the combined relative distribution of OTU 06 and OTU 09 (*Alphaproteobacteria*) was 22% in the SF but < 0.1% in the SS scheme (Supplementary Figure S2). Detailed information of the relative distributions of functional groups (e.g., AOB, NOB) identified at each operational scheme can be found in Gomez-Alvarez et al. (2016).

**TABLE 2:** Results of One-way PERMANOVA test based on Jensen–Shannon dissimilarity matrix derived from the distribution of microbial communities.

Source of Variation	df	Sum of Squares	Mean Square	Pseudo-*F* value	*P* value	Permutations
Operational scheme^‡^	1	21,602	21,602	17.47	< 0.0001	9915
Residual	38	46,997	1,237			
Total	39	68,600				

*^‡^Operational schemes: SS, stable chloramine residual; SF, complete nitrification and minimal chloramine residual. ^†^Significance set at α = 0.01.*

Linear discriminative analysis effect size (LEfSe) with stringent conditions (LDA = 4.0, *p* < 0.01) identified 70 and 62 OTU-level bioindicators in the SF and SS operational schemes, respectively. Furthermore, LEfSe identified 144 discriminative genus-level taxonomic groups (45 and 99 in the SF and SS, respectively).

### Model-Building and Evaluation

We utilized a machine learning approach (naïve Bayes classifier) with biomass (ATP) and microbiome datasets as potential bioindicators in detecting failed DWDS experiencing nitrification episodes. First, a total of eight OTU-level bioindicators with the highest LDA score were selected for evaluation to avoid overfitting of the model (Figure 3B). A similar approach was used with the genus-level taxonomic groups (Supplementary Table S2), with the three genus-level bioindicators overrepresented (Taxa B1–B3), while five were underrepresented (Taxa A1–A5) in the SS scheme (Supplementary Table S3 and Figure S4A).

Performance of each classification model was evaluated using the AUC from the ROC and PR curves. ROC-AUC and PR-AUC using biomass data were determined to be 0.477 (CI 0.274–0.680) and 0.553 (CI 0.351–0.755), respectively (Figures 3C,D); which is equivalent to a random classification of the subjects. The values gradually increased when genus-level taxonomic membership (i.e., presence and absence) data were used in the classification model (ROC-AUC = 0.684, CI 0.533–0.835; PR-AUC = 0.689, CI 0.539–0.839; Supplementary Figures S4B,C). We observed that using OTU-level membership, the ROC-AUC and PR-AUC increased significantly to 0.871 (CI 0.767–0.975, *p* < 0.05) and 0.861 (CI 0.754–0.968, *p* < 0.05), respectively (Figures 3C,D). Furthermore, combining membership with distribution (i.e., community structure) for both the genus-level taxonomic and OTU-level sets significantly improved the predictive ability of the model beyond that of membership only (ROC-AUC and PR-AUC = 1.000, *p* < 0.01; Figures 3C,D and Supplementary Figures S3B,C).

### Classification Performance on Full-Scale DWDS Test Sets

We subsequently performed classification analysis on full-scale DWDS genus-level taxonomic test sets of *categorized* (Supplementary Table S4) and *uncategorized* instances (Supplementary Table S5). First, we tested the hypothesis that the reference database used to assign taxonomic affiliation (i.e., features) could affect the ability of our model to classify BW samples. The performance of the model on an RDP-generated test set of *categorized* instances resulted in higher ROC-AUC and PR-AUC values (0.825 and 0.821, respectively), corresponding to a sensitivity of 0.729 and a specificity of 0.894 (Supplementary Figure S5). The PPV and negative predictive value (NPV) were 0.745 and 0.886, respectively. The microbiome-based model correctly classified 136 (out of 161) instances (Table 3). Next, the test sets using the SILVA reference database resulted in similar AUC values; however, the use of the Greengenes dataset yielded lower AUCs (< 0.681), with a sensitivity of 0.229 and a specificity of 0.885 (Supplementary Figure S5). Further analysis and comparisons were performed using RDP-based test sets.

**TABLE 3:** Evaluation results for the prediction of bulk water (BW) samples.

Location (References)	*N*	Operational Scheme^‡^			Classification^†^		Classified		
		SS	SF	Uncategorized	SS	SF	Correctly	Incorrectly	Error (%)
**Western Australia** (Shaw et al., 2015)	13	10	3	–	10	3	13	0	0.0
**DWDS simulator** (this study)	11	11	0	–	10	1	10	1	9.1
**Pinellas County, FL** (Wang et al., 2014)	137	92	45	–	93	44	114	23	16.8
**Arkansas and Mississippi** (Holinger et al., 2014)	21	–	–	21	17	4	–	–	–
**Ohio River basin** (Stanish et al., 2016)	9	–	–	9	7	2	–	–	–
**Ann Arbor, MI** (Pinto et al., 2014)	116	–	–	116	98	18	–	–	–
**Eastern United States** (Ji et al., 2015)	30	–	–	30	26	4	–	–	–
**New Orleans, LA** (Hull et al., 2017)	40	–	–	40	40	0	–	–	–
**Urbana, IL** (Hwang et al., 2012)	14	–	–	14	14	0	–	–	–

*^‡^Operational schemes: SS, stable chloramine residual; SF, complete nitrification and minimal chloramine residual. ^†^Naïve Bayes Taxa-based classification model. DWDS, drinking water distribution systems.*

**FIGURE 4:**
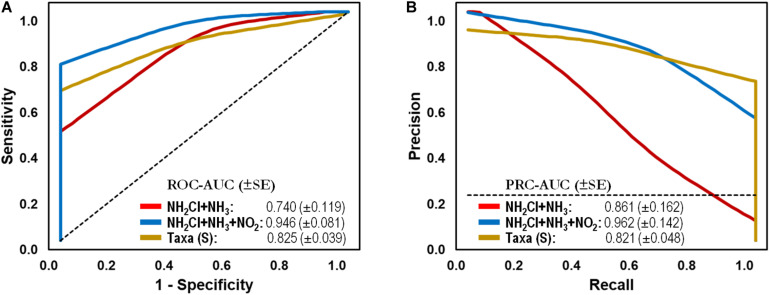
Classification performance on full-scale drinking water distribution systems (DWDS). **(A)** Receiver operating characteristic (ROC) and **(B)** Precision-recall (PR) curves with area under the curve (AUC) values and 95% confidence intervals in parenthesis for predictive model comparing microbial bioindicators based on community structure (genus-level taxonomy: RDP taxonomic database, 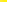) and water quality (parameters: NH_2_Cl + NH_3_, 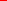; NH_2_Cl + NH_3_ + NO_2_^–^, 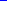) data. Dashed lines indicate the null model. Samples: Failure, *n* = 48; Stable, *n* = 113.

To test the capacity of the water microbiome as a screening tool, we generated classification models using two common chemical parameters used for monitoring water quality in DWDS. In comparison, an abiotic-based model using total Chlorine/NH_2_Cl and NH_3_ generated lower AUCs of 0.740 and 0.861 (ROC and PR, respectively), corresponding to a sensitivity of 0.250 and a specificity of 0.957. The AUCs increased to > 0.946 with the addition of NO_2_^–^ concentration (i.e., feature) to the test set, which is indicative of nitrification (> 0.05 mg/L, i.e., Failure) in the DWDS. As can be seen in Figure 4, the microbiome-based model significantly improved the ability to distinguish between Stable and Failure instances compared with the model containing disinfectant residual and ammonia.

Finally, we explored the ability of our microbiome-based model to classify a test set of *uncategorized* instances (*n* = 230). A total of 202 instances were classified as Stable samples while the remainder twenty-eight contained microbial communities associated with a Failure system (Table 3). In comparison, a decrease in the classification of failed system (nine instances) was observed when an abiotic-based model (total Chlorine/NH_2_Cl and NH_3_) was used in the analysis. Further evaluation was impossible since most of the published research studies used in this study omitted the operational status of the DWDS or did not include key water chemical parameters of nitrification (e.g., NO_2_^–^).

## Discussion

In this study, we report significant differences in the microbial structure of BW samples and demonstrate the potential of water microbiome profiles as bioindicators for system stability in DWDS. We also observed the occurrence and dominance of specific microbial groups (*Proteobacteria* and *Actinobacteria*) which is consistent with other DWDS studies (Hwang et al., 2012; Holinger et al., 2014; Pinto et al., 2014; Wang et al., 2014; Ji et al., 2015; Shaw et al., 2015; Stanish et al., 2016; Hull et al., 2017), albeit not at the same ratios. In addition, a previous study by Gomez-Alvarez et al. (2016) demonstrated that the microbial communities associated with DWDS were sensitive to changes in operational parameters (e.g., temperature, disinfectant residual) and responded to a disturbance by returning to a stable state after a shift in community composition (i.e., resilience). Potential microbiome bioindicator candidates for the monitoring of DWDS met two key factors: (i) measurable changes in the relative distribution of specific microbial groups during episodes of disturbance (Gomez-Alvarez et al., 2016), and (ii) the impact the water microbiome has on water quality (Zhang et al., 2009). Bioindicators are by definition objective and quantifiable biological features (i.e., living organisms) that can be used to indicate the quality and stability of their ecosystem (Siddig et al., 2016), and can be measured at higher levels of organization (e.g., species, population, community; McCarty et al., 2002; Bartell, 2006). Fundamentally, the goal of bioindicators/biomarkers development is to build a predictive model from a collection of biological data (i.e., features) and a qualitative dependent variable, which can be used to classify/predict new instances into specific categories with optimal sensitivity and specificity (Xia et al., 2013).

Recent advances in ‘omics’ technologies and bioinformatics for systems analyses have revitalized the field of bioindicator/biomarker discovery. However, the unprecedented amount of data makes the goal of building predictive models from metagenomic studies difficult to obtain with traditional approaches. To cope with this complexity, machine learning techniques and supervised classification have recently been suggested as a promising tool useful for recognizing patterns in complex datasets derived from metagenomic studies (Libbrecht and Noble, 2015). The use of such techniques for bioindicator discovery and prediction has been explored by several researchers although mostly in clinical studies. For example, sequencing methods targeting 16S rRNA regions have allowed identification of microbiome-associated bioindicators that correlate with protection against CTLA-4 blockade-associated colitis (Dubin et al., 2016), presence of adenomatous polyps in patients (Hale et al., 2017), to differentiate patients with *Clostridium difficile* infection and non-*C. difficile*-associated diarrhea from healthy controls (Schubert et al., 2014) and for the identification of individuals harboring adenomas and carcinomas (Zackular et al., 2014). Overall, machine learning may hold a promising future in predictive modeling for metagenome based DWDS studies.

The steps involved in predictive modeling are bioindicator selection, performance evaluation and classification model creation. In the case of bioindicator selection, our study identified 132 OTU-level discriminative bioindicators, but only eight OTU-level bioindicators with the highest LDA score were selected for evaluation to avoid overfitting of the model. Overfitting occurs every time an algorithm excessively adapts to the training set. The selected OTU-level bioindicators correspond to three bioindicators overrepresented and five bioindicators underrepresented in the Stable (SS) operational scheme (Supplementary Figure S2). The overrepresented OTUs (i.e., genus-level taxonomic group) are members of the class *Proteobacteria* (family *Rhizobiales* and *Sphingomonadales*) and *Actinobacteria* (*Mycobacterium*), while the underrepresented are representatives of the *Nitrospira* (NOB) and *Proteobacteria* [*Nitrosomonas* (AOB) and *Sphingomonadales*] (Supplementary Table S2). Like other published studies, *Actinobacteria* shared similar patterns of occurrence in chloraminated systems despite their geographic separation, which was demonstrated to be characteristic of this drinking water environment (Wang et al., 2012; Bautista-de los Santos et al., 2016). Furthermore, a meta-analysis of several 16S rRNA gene sequencing studies corroborated the enrichment and depletion of these bacterial populations in full-scale DWDS which were associated with changes in water quality (Bautista-de los Santos et al., 2016). Furthermore, Gomez-Alvarez et al. (2016) reiterated the common notion that disturbances induce a strong selection pressure on microbial populations. In conclusion, these findings stress the importance of using not just one single bacterial population, but rather a consortium of bacterial populations, in predicting the stability of the DWDS.

Nitrification is triggered by the release of NH_3_ during chloramine decay and is accomplished through a two-step microbiological process; the oxidation of NH_3_ to NO_2_^–^ by AOB follow by the oxidation of NO_2_^–^ to NO_3_^–^ carried out by NOB. Both groups of nitrifying bacteria are ubiquitously distributed in chloraminated DWDS. As indicated previously, a group of AOB and NOB were overrepresented in the Failure (SF) operational scheme samples (Supplementary Table S2). The representation and predominance of AOB and NOB populations during nitrification is consistent with a previously reported molecular analysis of the population in a pilot scale chloraminated distribution system (Regan et al., 2002). In a recent study, quantitative PCR analysis indicated higher frequency of AOB and NOB populations detected before the chlorine burn on a failed DWDS, while the presence of these two groups was greatly reduced after the chlorine burn (Wang et al., 2014). Changes in the microbial community associated with nitrification may represent early events in the pathway leading to failed systems. This has considerable importance in developing monitoring plans to predict future nitrification episodes in water systems compared to current methods which only confirm that nitrification is occurring. Although our results are supported by previous evidence, further studies in full-scale DWDS are needed to determine whether these bacterial populations indeed are found more frequently in failed full-scale chloraminated DWDS and are associated with nitrification exclusively.

We developed a naïve Bayes classification model to discriminate operational schemes (SS and SF events) based on the selection of eight OTU-level bioindicators (i.e., training set). First, incorporation of the membership (i.e., presence and absent) to the set provided a marginal improvement (AUC < 0.689) in classification versus a biomass-based model (i.e., ATP: AUC < 0.553). In line with other studies (Schubert et al., 2014; Zackular et al., 2014), the inclusion of the relative abundance data significantly improved the predictive ability of the model beyond that of membership only (AUC = 1.000). Furthermore, similar performance was obtained using the training set based on the relative abundance at genus-level taxonomic affiliation of the eight bioindicators (i.e., Taxa-level training set). Inclusion of the relative abundances significantly enhanced the ability to discriminate BW samples regardless of the type of data [OTU (sequence-based) or taxa (taxonomy-based)] we used to represent the microbiome. Moreover, we found that the latter approach has the advantages that it is not limited by the exhaustive computation required for OTU-based analysis (e.g., alignment and clustering), is more tolerant of sequencing errors, and allows comparisons when sequences are from different regions of the 16S rRNA gene (Sul et al., 2011).

The naïve Bayes Taxa-based classification model was able to correctly classify 85% of *categorized* instances (documented nitrification or stable events) with an AUC of 0.825 (sensitivity: 0.729, specificity: 0.894) on a full-scale DWDS test set (Table 3). In comparison, abiotic-based model using total Chlorine/NH_2_Cl and NH_3_ generated AUCs of 0.740 and 0.861 (ROC and PR, respectively), corresponding to a sensitivity of 0.250 and a specificity of 0.957. The AUCs increased to > 0.946 with the addition of NO_2_^–^ concentration, which is indicative of nitrification in the DWDS. This research provides evidence of the feasibility of using bioindicators to predict operational failures in the system (e.g., nitrification). The discriminative power of the model was not influenced by geographical location or sequencing technology used in the DWDS study, reinforcing the potential of the microbiome as a capable classification tool of system stability in DWDS. Although established protocols of monitoring reported the association between water parameters and/or culture-based assays with system stability (Hill and Arweiler, 2013), our supervised machine learning analysis provided a significant improvement in the classification of BW samples. Microbial populations are directly involved in the process of nitrification, which make them perfect bioindicator candidates for the prediction of future nitrification episodes as current monitoring plans only confirm that nitrification is occurring. Furthermore, our analysis demonstrated the importance of reference database selection for the taxonomic classification of populations in BW samples (i.e., test sets). For example, the use of the Greengenes-based test set yielded a decrease in sensitivity and specificity in comparison to the RDP- and SILVA-based test sets. The discrepancy in performance against the latter reference databases may be explained by the fact that Greengenes is the smallest reference database with much less diversity than the other taxonomies, and it has not been updated for the last 4 years (Balvočiūtė and Huson, 2017).

It is important to mention the research challenges and opportunities faced during our study. First, of the publicly available BW samples used in this study for evaluation, up to 59% were *uncategorized* samples. These chloraminated DWDS metagenome-based studies contain incomplete information related to water parameters and stability of the system. Therefore, we used our classification model to predict the operational schemes of these samples (Table 3). Most of the samples were classified as SS (88% out of 230) with the rest 12% as SF. Nonetheless, these results will be valuable for utilities about the potential of microbial communities associated with nitrification at these locations in their DWDS. However, without a complete categorization of the system, we cannot confirm the actual operational status (stable or failure) of these samples. Next, our models were based on the water microbiome at two very specific operational schemes (Hill and Arweiler, 2013). Our study was designed to reproduce a catastrophic event in a DWDS. Thus, we are limited in our ability to identify the incremental progressive changes in the water microbiome toward nitrification or the recovery to a stable state. Again, the aim of this study was to identify components of the microbiome that are associated with SS and SF. Description of such changes will be possible only if BW samples are routinely collected before and after the development of a nitrification event. Finally, understanding the response of microbial communities in disturbed DWDS environments is essential for risk management and for evaluating the biological stability of the systems. Such information could be incorporated into ecosystem process models and greatly enhance our ability to predict ecosystem responses to disturbances. For example, supervised machine learning can be used to build a model from a set of categorized data points that can classify/predict the correct category of unlabeled data, whenever alternative methods for obtaining data classification are difficult (Knights et al., 2011).

## Conclusion

We report here evidence of the feasibility of using microbiome-based bioindicators to predict operational failures in the system (e.g., nitrification). While the method requires additional validation and is not deployable in the presented form, it could be developed as a portable quantitative tool to detect bioindicators in the distribution system. These bioindicators may reveal the potential onset of a nitrification episode before sufficient levels of chemical surrogates are detected. Traditional methods of culturing, e.g., AOB MPN requires weeks of incubation and they lack the ability to detect AOBs in the DWDS when they occur at low levels. Importantly, the discriminative power of the model was not influenced by geographical location, disinfectant treatment or sequencing technology used to generate the microbiome profile. Unlike chemical surrogates, bioindicators monitor the microbial populations responsible for the chemical surrogates during onset of nitrification. Previous studies recognized the correlation between nitrification and an early warning indicator, i.e., surrogate (for details see Regan et al., 2002; Pintar et al., 2005). The information generated in this study improves our understanding of the drinking water ecosystem, particularly the unintended consequences of disinfectant switching practices and how bioindicators may be a screening tool and a potential predictor of nitrification in DWDS.

## Data Availability Statement

The datasets generated in this study can be found in online repositories. The names of the repository/repositories and accession number(s) can be found below: https://trace.ncbi.nlm.nih.gov/Traces/sra/?study=SRP069876.

## Author Contributions

VG-A and RR designed the study and performed the research. VG-A analyzed the data, constructed the model, and drafted the manuscript. Both authors contributed to the final manuscript.

## Disclaimer

The authors declare that the research was conducted in the absence of any commercial or financial relationships that could be construed as a potential conflict of interest.

## Conflict of Interest

The authors declare that the research was conducted in the absence of any commercial or financial relationships that could be construed as a potential conflict of interest.

## Footnotes

1 https://www.mothur.org

2 http://huttenhower.org/galaxy
