# Supplementary material for: Monitoring of Nitrification in Chloraminated Drinking Water Distribution Systems With Microbiome Bioindicators Using Supervised Machine Learning
Source: Front Microbiol. 2020 Sep 16;11:571009. doi: 10.3389/fmicb.2020.571009 (PMC7526508; doi:10.3389/fmicb.2020.571009)
Supplement: Supplementary file 1 [file Image_1.PDF]

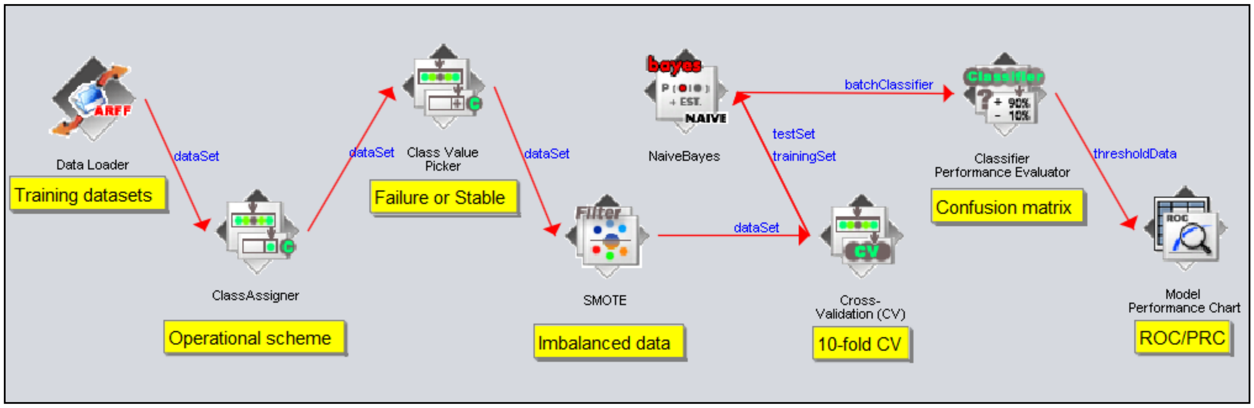

Figure S1. Machine Learning Classification and Evaluation. WEKA Knowledge Flow Interface steps to generate the classification model using a naïve Bayes supervised classification algorithm.
