# Supplementary material for: Monitoring of Nitrification in Chloraminated Drinking Water Distribution Systems With Microbiome Bioindicators Using Supervised Machine Learning
Source: Front Microbiol. 2020 Sep 16;11:571009. doi: 10.3389/fmicb.2020.571009 (PMC7526508; doi:10.3389/fmicb.2020.571009)
Supplement: Supplementary file 2 [file Image_2.PDF]

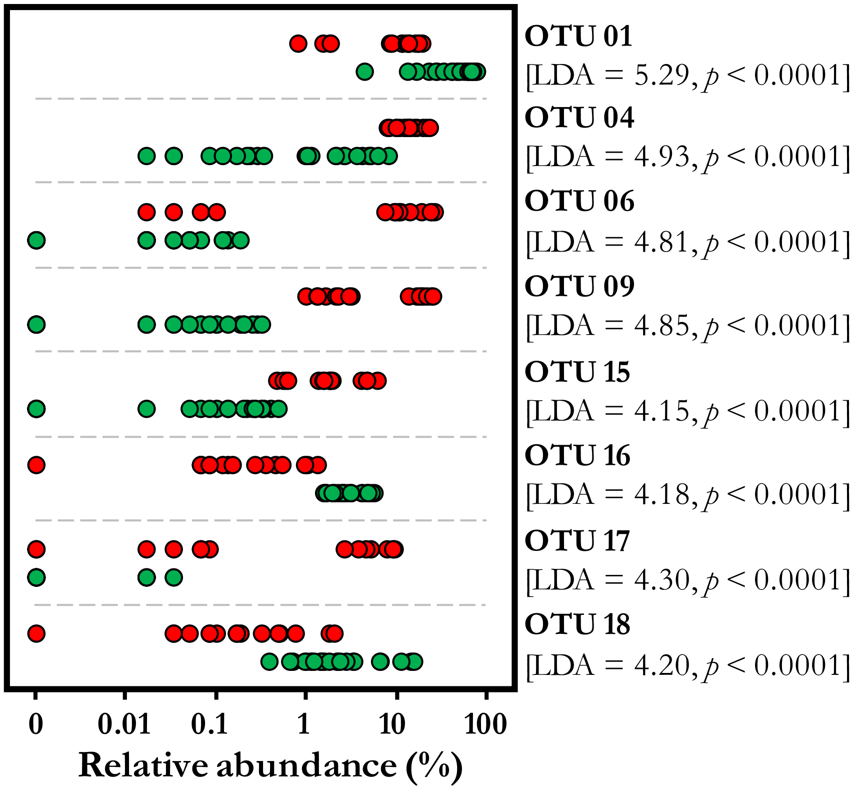

Figure S2. OTU-level assigned bioindicators. Relative abundance of differentially abundant OTU-level assigned bioindicators using linear discriminative analysis (LDA) effect size (LEfSe) analyses (LDA score > 4.0, $p < 0.0001$). Samples: Stable (SS, ●) and Failure (SF, ●) events.
