# Supplementary material for: Monitoring of Nitrification in Chloraminated Drinking Water Distribution Systems With Microbiome Bioindicators Using Supervised Machine Learning
Source: Front Microbiol. 2020 Sep 16;11:571009. doi: 10.3389/fmicb.2020.571009 (PMC7526508; doi:10.3389/fmicb.2020.571009)
Supplement: Supplementary file 3 [file Image_3.PDF]

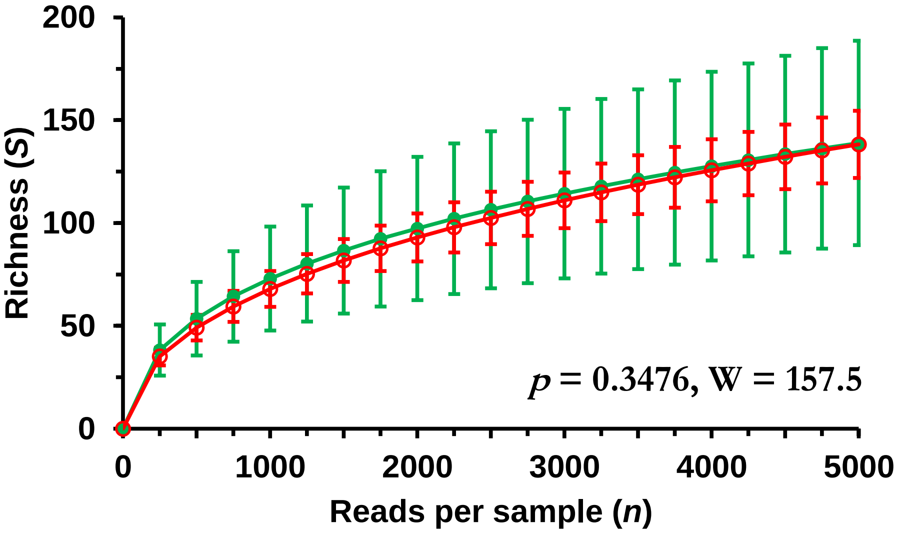

Figure S3. Microbial diversity. Rarefaction curves for the DWDS bulk water microbiomes. For comparison, specie frequencies were normalized to the smallest library (n = 5 000). The p value was calculated by the Wilcoxon-Mann-Whitney test. Significance set at α = 0.01. Samples: Stable (SS, ●), Failure (SF, ●) events.
