# Supplementary material for: Monitoring of Nitrification in Chloraminated Drinking Water Distribution Systems With Microbiome Bioindicators Using Supervised Machine Learning
Source: Front Microbiol. 2020 Sep 16;11:571009. doi: 10.3389/fmicb.2020.571009 (PMC7526508; doi:10.3389/fmicb.2020.571009)
Supplement: Supplementary file 4 [file Image_4.PDF]

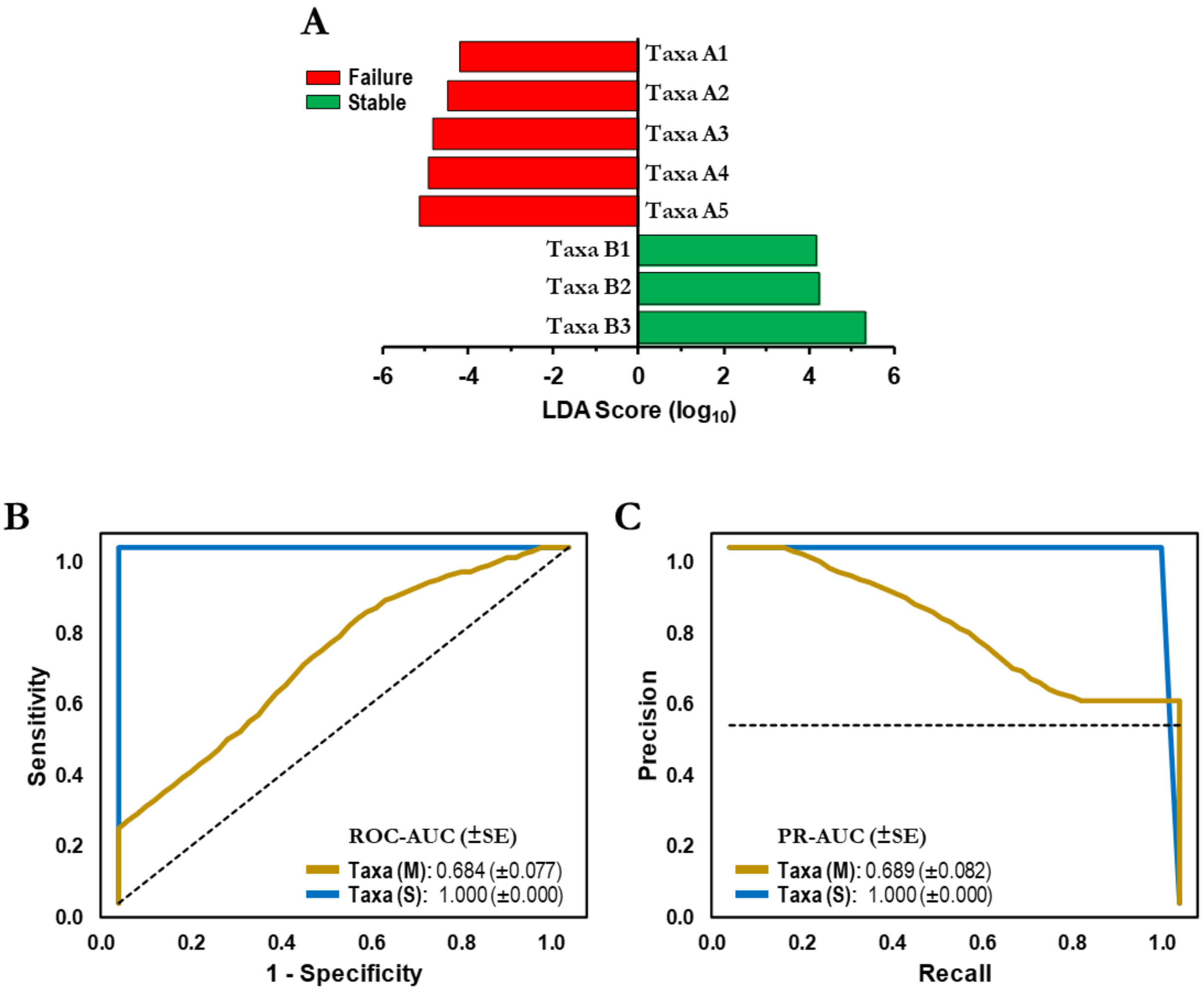

Figure S4. Genus-level taxonomic assigned bioindicators and classification performance. (A) Identification of statistically significant genus-level taxonomic groups using linear discriminative analysis (LDA) effect size (LEfSe) analyses (LDA score > 4.0, $p < 0.01$). Negative LDA scores are enriched in SF while positive LDA scores are enriched in SS events. Samples: Stable (SS, ●), Failure (SF, ●) events. (B) Receiver operating characteristic (ROC) and (C) Precision-recall (PR) curves with AUC values and 95% confidence intervals in parenthesis for predictive model comparing microbial bioindicators based on community membership (▬) and structure (▬) data. Dashed lines indicate the null model.
