# Supplementary material for: Monitoring of Nitrification in Chloraminated Drinking Water Distribution Systems With Microbiome Bioindicators Using Supervised Machine Learning
Source: Front Microbiol. 2020 Sep 16;11:571009. doi: 10.3389/fmicb.2020.571009 (PMC7526508; doi:10.3389/fmicb.2020.571009)
Supplement: Supplementary file 5 [file Image_5.PDF]

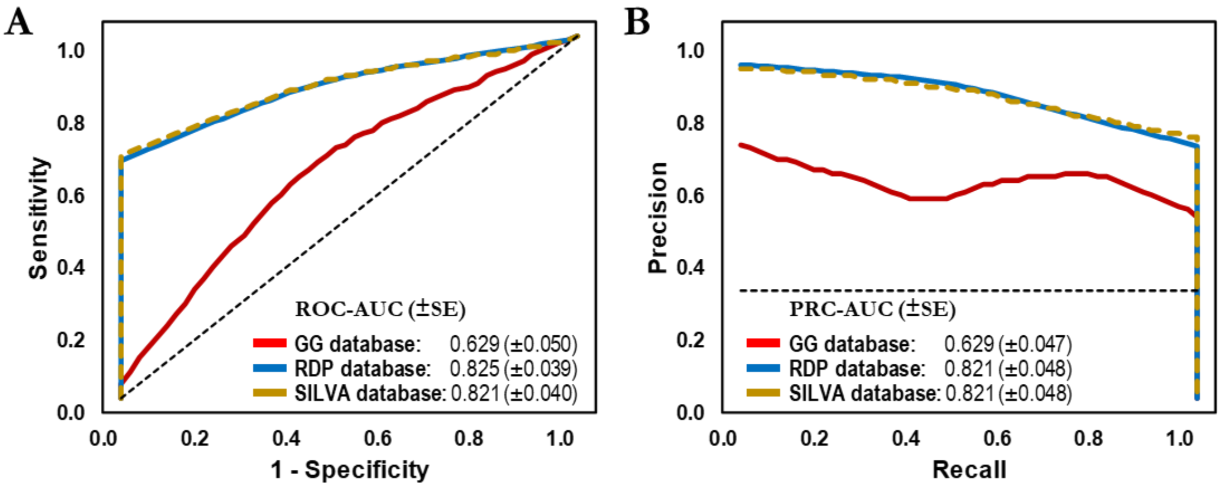

Figure S5. Comparison of genus-level taxonomic identification. (A) Receiver operating characteristic (ROC) and (B) Precision-recall (PR) curves with AUC values and 95% confidence intervals in parenthesis for predictive model comparing microbial bioindicators based on community structure using genus-level taxonomy. Taxonomic classification was determined using the GG (—), RDP (—) and SILVA (—) reference taxonomic databases. Dashed lines indicate the null model.
