# Supplementary material for: Monitoring of Nitrification in Chloraminated Drinking Water Distribution Systems With Microbiome Bioindicators Using Supervised Machine Learning
Source: Front Microbiol. 2020 Sep 16;11:571009. doi: 10.3389/fmicb.2020.571009 (PMC7526508; doi:10.3389/fmicb.2020.571009)
Supplement: Supplementary file 6 [file Table_1.PDF]

Table S1. Water quality values (±SD) for operational schemes in the pipe-loop system*.

Characteristics	Stable[‡] SS (n = 11)	Failure SF (n = 5)	P value[†]	Restore SR (n = 7)
**Parameters**				
pH	7.88 ± 0.11	7.80 ± 0.11	0.1889	7.77 ± 0.04
turbidity [NTU]	0.36 ± 0.48	0.10 ± 0.02	0.6797	0.37 ± 0.34
**Disinfectant**				
NH_2Cl [Cl_2 mg L^{-1}]	1.03 ± 0.43	0.06 ± 0.06	0.0005	0.01 ± 0.00
Free Chlorine [Cl_2 mg L^{-1}]	0.01 ± 0.01	0.01 ± 0.00	0.9999	2.57 ± 0.66
**C, N & P compounds**				
Ammonia-Nitrogen [mg L^{-1}]	0.20 ± 0.08	0.03 ± 0.01	0.0080	0.02 ± 0.03
Nitrite-Nitrogen [mg L^{-1}]	0.01 ± 0.01	0.22 ± 0.05	0.0005	0.00 ± 0.00
Nitrate-Nitrogen [mg L^{-1}]	1.02 ± 0.20	0.91 ± 0.18	0.2211	0.92 ± 0.04
Phosphate [mg L^{-1}]	0.15 ± 0.04	0.21 ± 0.06	0.0408	0.07 ± 0.03
Total Organic Carbon [mg L^{-1}]	0.78 ± 0.21	0.63 ± 0.15	0.0686	0.93 ± 0.06
Total Nitrogen [mg L^{-1}]	1.36 ± 0.25	1.25 ± 0.19	0.5673	0.96 ± 0.05
C/N ratio	0.59 ± 0.17	0.52 ± 0.17	0.5096	0.96 ± 0.07

[*]Pipe-loop properties: 150 mm gray schedule 80 PVC; 0.30 m s^{-1} recirculation flow rate; 27 m length.

[‡]Operational schemes: **SS =** stable chloramine residual; **SF =** complete nitrification and minimal chloramine residual; **SR =** chlorine burn.

[†]Mann-Whitney U test between Stable (SS) and Failure (SF) at $\alpha = 0.01$.
