# Supplementary material for: Monitoring of Nitrification in Chloraminated Drinking Water Distribution Systems With Microbiome Bioindicators Using Supervised Machine Learning
Source: Front Microbiol. 2020 Sep 16;11:571009. doi: 10.3389/fmicb.2020.571009 (PMC7526508; doi:10.3389/fmicb.2020.571009)
Supplement: Supplementary file 7 [file Table_2.PDF]

Table S2. Relative distribution of genus-level taxonomic bioindicators generated from a DWDS simulator. Values represent abundance (±SD) of each taxon as a ratio of all sequences obtained at each operational scheme.

**Taxonomy groups** (bioindicators)	**Lineage**	**Stable**[‡]	**Failure**
		**SS** (n = 24)	**SF** (n = 16)
**Taxa A1**	*Nitrospira*	0.0022 ± 0.0013	0.0220 ± 0.0171
**Taxa A2**	*Nitrosomonas*	0.0046 ± 0.0047	0.0495 ± 0.0383
**Taxa A3**	*Erythromicrobium*	0.0012 ± 0.0010	0.1079 ± 0.0950
**Taxa A4**	*Sphingomonas*	0.0228 ± 0.0244	0.1391 ± 0.0446
**Taxa A5**	*Hyphomicrobium*	0.0053 ± 0.0034	0.2217 ± 0.1733
**Taxa B1**	*Bradyrhizobium*	0.0311 ± 0.0129	0.0038 ± 0.0040
**Taxa B2**	*Sphingopyxis*	0.0365 ± 0.0431	0.0044 ± 0.0063
**Taxa B3**	*Mycobacterium*	0.4877 ± 0.2137	0.1071 ± 0.0612

[‡]Operational schemes: **SS =** stable chloramine residual; **SF =** complete nitrification and minimal chloramine residual
