# Supplementary material for: Monitoring of Nitrification in Chloraminated Drinking Water Distribution Systems With Microbiome Bioindicators Using Supervised Machine Learning
Source: Front Microbiol. 2020 Sep 16;11:571009. doi: 10.3389/fmicb.2020.571009 (PMC7526508; doi:10.3389/fmicb.2020.571009)
Supplement: Supplementary file 8 [file Data_Sheet_1.PDF]

Table S3. Training set based on genus-level taxonomic bioindicators generated from a DWDS simulator. Values represent abundance of each taxon as a ratio of all sequences obtained for each individual sample. Legend: ND = not detected.

**Sample** (reference)	**Operational scheme**	**Taxonomy groups** (bioindicators)							
		**Taxa A1**	**Taxa A2**	**Taxa A3**	**Taxa A4**	**Taxa A5**	**Taxa B1**	**Taxa B2**	**Taxa B3**
**A13_03_20_1**	Stable	0.0013	ND	0.0020	0.0012	0.0098	0.0465	0.1462	0.2775
**B13_03_20_1**	Stable	ND	ND	0.0003	0.0002	0.0015	0.0195	0.0227	0.0443
**A13_03_20_2**	Stable	0.0022	0.0007	0.0028	0.0052	0.0095	0.0465	0.1575	0.2267
**B13_03_20_2**	Stable	ND	ND	ND	0.0003	0.0017	0.0155	0.0337	0.2710
**B13_05_15_1**	Stable	0.0007	0.0010	0.0007	0.0028	0.0053	0.0308	0.0163	0.1657
**A13_05_15_2**	Stable	0.0040	0.0033	0.0010	0.0117	0.0025	0.0405	0.1125	0.4050
**B13_05_15_2**	Stable	ND	ND	0.0005	0.0023	0.0047	0.0285	0.0153	0.1327
**A13_06_12_1**	Stable	0.0033	0.0153	0.0010	0.0507	0.0057	0.0253	0.0670	0.4583
**B13_06_12_1**	Stable	0.0048	0.0095	0.0018	0.0430	0.0055	0.0247	0.0660	0.4753
**A13_06_12_2**	Stable	0.0032	0.0087	0.0007	0.0523	0.0045	0.0413	0.0357	0.5587
**B13_06_12_2**	Stable	0.0005	0.0038	0.0008	0.0273	0.0048	0.0560	0.0185	0.3305
**A14_03_05_1**	Stable	ND	ND	0.0025	0.0582	0.0083	0.0162	0.0072	0.6093
**B14_03_05_1**	Stable	ND	ND	0.0013	0.0377	0.0098	0.0250	0.0190	0.5055
**A14_03_05_2**	Stable	ND	ND	0.0032	0.0853	0.0132	0.0260	0.0143	0.4133
**B14_03_05_2**	Stable	ND	0.0002	0.0020	0.0625	0.0117	0.0205	0.0132	0.6768
**A14_04_15_1**	Stable	0.0002	0.0005	0.0002	0.0227	0.0048	0.0155	0.0117	0.7442
**B14_04_15_1**	Stable	0.0010	0.0003	0.0002	0.0035	0.0058	0.0192	0.0113	0.7723
**A14_07_30_1**	Stable	0.0027	0.0143	ND	0.0278	0.0035	0.0165	0.0222	0.6703
**B14_07_30_1**	Stable	0.0025	0.0050	ND	0.0107	0.0020	0.0522	0.0240	0.6175
**A14_07_30_2**	Stable	0.0032	0.0035	ND	0.0247	0.0032	0.0225	0.0115	0.7200
**B14_07_30_2**	Stable	0.0027	0.0047	ND	0.0110	0.0022	0.0478	0.0123	0.6985
**A14_09_17_1**	Stable	0.0023	0.0038	0.0003	0.0042	0.0033	0.0308	0.0278	0.6070
**B14_09_17_1**	Stable	0.0008	0.0013	0.0002	0.0010	0.0018	0.0478	0.0038	0.6528
**B14_09_17_2**	Stable	0.0013	0.0017	ND	0.0018	0.0010	0.0307	0.0070	0.6715
**A13_08_07_1**	Failure	0.0047	0.0190	0.1658	0.1288	0.0140	0.0102	0.0185	0.1900
**B13_08_07_1**	Failure	0.0057	0.0377	0.1972	0.1080	0.0072	0.0045	0.0078	0.1732
**A13_08_07_2**	Failure	0.0058	0.0167	0.1348	0.1202	0.0105	0.0132	0.0207	0.1375
**B13_08_07_2**	Failure	0.0062	0.0147	0.1787	0.1560	0.0107	0.0095	0.0052	0.1745
**A13_09_04_1**	Failure	0.0135	0.0390	0.2393	0.1267	0.2290	0.0007	0.0033	0.1165
**B13_09_04_1**	Failure	0.0152	0.0123	0.2155	0.1392	0.3203	0.0053	0.0012	0.0848

**A13_09_04_2**	Failure	0.0192	0.0277	0.1878	0.1412	0.2942	0.0013	0.0018	0.1157
**B13_09_04_2**	Failure	0.0182	0.0137	0.2500	0.1383	0.2813	0.0017	0.0010	0.0895
**A13_10_23_1**	Failure	0.0610	0.0548	0.0098	0.1690	0.1282	0.0012	0.0055	0.1638
**B13_10_23_1**	Failure	0.0463	0.0387	0.0132	0.2122	0.1742	0.0035	0.0017	0.1297
**A13_10_23_2**	Failure	0.0405	0.0485	0.0163	0.1942	0.1257	0.0007	0.0008	0.1353
**B13_10_23_2**	Failure	0.0465	0.0292	0.0210	0.2323	0.1340	0.0027	0.0017	0.1372
**A13_11_14_1**	Failure	0.0180	0.0970	0.0313	0.0980	0.4637	0.0007	0.0003	0.0180
**B13_11_14_1**	Failure	0.0185	0.1113	0.0227	0.0837	0.4750	0.0008	0.0002	0.0157
**A13_11_14_2**	Failure	0.0177	0.1148	0.0130	0.0787	0.5017	ND	0.0005	0.0128
**B13_11_14_2**	Failure	0.0157	0.1163	0.0297	0.0990	0.3778	0.0008	0.0003	0.0187
