# Supplementary material for: Monitoring of Nitrification in Chloraminated Drinking Water Distribution Systems With Microbiome Bioindicators Using Supervised Machine Learning
Source: Front Microbiol. 2020 Sep 16;11:571009. doi: 10.3389/fmicb.2020.571009 (PMC7526508; doi:10.3389/fmicb.2020.571009)
Supplement: Supplementary file 9 [file Data_Sheet_2.PDF]

Table S4. Test set based on genus-level taxonomic bioindicators of *categorized* instances (i.e. operational parameters) obtained from a DWDS simulator and full-scale DWDS studies. Values represent abundance of each taxon as a ratio of all sequences obtained for each individual sample. Legend: ND = not detected; missing value = ?. †Sequence data was reanalyzed using RDP v16 database.

**Sample** (reference)	**Operational scheme**	**Taxonomy groups** (bioindicators)							
		**Taxa A1**	**Taxa A2**	**Taxa A3**	**Taxa A4**	**Taxa A5**	**Taxa B1**	**Taxa B2**	**Taxa B3**
Western Australia (**Shaw et al., 2015**)									
**SA PT 1.5**	Stable	ND	?	ND	?	ND	?	?	ND
**SA-1 CT**	Failure	0.0121	?	ND	?	0.0001	?	?	0.0020
**SA-2**	Stable	0.0003	?	ND	?	ND	?	?	ND
**SA-2 CT**	Failure	0.0284	?	ND	?	ND	?	?	ND
**SA-3**	Stable	0.0002	?	ND	?	ND	?	?	ND
**SA-4**	Stable	ND	?	ND	?	ND	?	?	ND
**SA-5 CT**	Stable	ND	?	ND	?	ND	?	?	0.0001
**WA-1A**	Stable	ND	?	ND	?	ND	?	?	ND
**WA-1B**	Stable	0.0008	?	ND	?	ND	?	?	ND
**WA-2**	Stable	0.0002	?	ND	?	ND	?	?	ND
**WA-3A**	Stable	0.0001	?	0.0001	?	ND	?	?	ND
**WA-3B**	Stable	ND	?	ND	?	ND	?	?	0.0003
**WA-4 CT**	Failure	0.0063	?	ND	?	ND	?	?	0.0064
DWDS simulator (this study)									
**A12_09_19_1**	Stable	0.0023	0.0005	ND	0.0013	0.0013	0.0120	0.1005	0.2293
**A12_10_01_1**	Stable	0.0010	0.0013	ND	0.0008	0.0028	0.0133	0.1293	0.3830
**A12_10_15_1**	Stable	ND	0.0013	ND	0.0008	0.0045	0.0158	0.1410	0.4383
**A12_10_23_1**	Stable	0.0003	0.0005	ND	0.0005	0.0063	0.0230	0.1005	0.6170
**A12_11_15_1**	Stable	ND	ND	0.0008	0.0038	0.0075	0.0283	0.1433	0.3705
**B12_09_19_1**	Stable	0.0003	0.0013	ND	0.0025	0.0065	0.0120	0.1048	0.2750
**B12_10_01_1**	Stable	0.0013	0.0013	ND	0.0005	0.0108	0.0140	0.1013	0.4135
**B12_10_15_1**	Stable	ND	0.0023	ND	0.0010	0.0043	0.0125	0.1210	0.3900
**B12_10_23_1**	Stable	0.0003	ND	ND	0.0010	0.0038	0.0045	0.0503	0.7813
**B12_11_08_1**	Stable	ND	ND	0.0273	0.0133	ND	0.0018	ND	0.1620
**B12_11_15_1**	Stable	0.0003	ND	0.0033	0.0078	0.0125	0.0298	0.0813	0.2498

Pinellas County, FL† (**Wang et al., 2014**)

**S1_PBR_001**	Failure	0.0278	0.0037	ND	0.0065	ND	ND	ND	0.0004
**S1_PBR_002**	Failure	0.0005	0.0015	ND	0.0006	ND	0.0003	ND	0.0005
**S1_PBR_003**	Failure	0.0846	0.0025	ND	0.0034	0.0033	0.0132	0.0013	0.0036
**S1_PBR_004**	Failure	0.0038	0.0828	ND	0.0009	0.0010	0.0006	0.0008	0.1962
**S1_PBR_005**	Failure	0.0004	0.0098	ND	0.0148	ND	0.0006	0.0225	0.0214
**S1_PBR_006**	Failure	0.0235	0.2109	ND	0.0254	0.0207	0.0030	0.0002	0.1409
**S1_PBR_007**	Failure	0.0123	0.0295	ND	0.0104	0.0025	0.0062	0.0018	0.0405
**S1_PBR_008**	Failure	0.0005	0.0967	ND	0.0001	0.0015	ND	ND	0.2239
**S1_PBR_009**	Failure	ND	0.0227	ND	0.0029	0.0215	ND	ND	0.0036
**S1_PBR_010**	Failure	0.0003	0.1814	ND	0.0039	0.0101	0.0002	0.0001	0.0595
**S1_PBR_011**	Failure	0.0001	0.0807	ND	0.0005	0.0003	0.0006	ND	0.0061
**S1_PBR_012**	Failure	0.0312	0.0038	ND	0.0094	0.0019	0.0035	0.0003	0.0015
**S1_PBR_013**	Failure	0.0022	0.2114	ND	0.0017	0.0061	0.0010	0.0003	0.0808
**S1_PBR_014**	Failure	0.0035	0.1072	ND	0.0597	0.0334	0.0013	0.0003	0.0613
**S1_PBR_015**	Failure	0.3359	0.0079	ND	0.0500	0.0024	0.0040	0.0505	0.0031
**S1_PBR_016**	Failure	0.0067	0.0321	ND	0.0009	0.0020	0.0007	0.0010	0.0024
**S1_PBR_017**	Failure	0.0042	0.0342	ND	0.1165	0.0076	0.0060	0.0012	0.0018
**S1_PBR_018**	Failure	0.0005	0.0364	ND	ND	ND	ND	0.0001	0.0089
**S1_PBR_019**	Failure	0.0042	0.2137	ND	0.0013	0.0105	0.0003	ND	0.0738
**S1_PBR_020**	Failure	0.0001	0.1213	ND	0.0002	0.0060	0.0001	0.0002	0.0438
**S1_PBR_021**	Failure	0.0004	0.0633	ND	0.0012	0.0072	0.0005	0.0007	0.0039
**S1_PBR_022**	Failure	0.0025	0.0490	ND	0.0083	0.0018	0.0004	0.0025	0.0214
**S1_PBR_023**	Failure	0.0014	0.0071	ND	0.0170	0.0003	0.0036	0.0062	0.0525
**S1_PBR_024**	Failure	0.0052	0.0575	ND	0.0020	0.0028	0.0020	0.0005	0.0094
**S1_PBR_025**	Failure	0.0007	0.0315	ND	0.0061	0.0004	0.0015	0.0045	0.0053
**S1_PBR_026**	Failure	0.0006	ND	ND	0.0021	0.0001	0.0003	0.0058	ND
**S1_PBR_027**	Failure	0.0012	0.0239	ND	0.0016	0.0049	0.0006	0.0001	0.0063
**S1_PBR_028**	Failure	0.0004	0.0207	ND	0.0075	0.0045	0.0002	0.0009	0.0096
**S1_PBR_029**	Failure	0.0004	0.0398	ND	0.0011	0.0038	0.0027	0.0015	0.0087
**S1_PBR_030**	Failure	0.0014	0.1136	ND	0.0047	0.0164	0.0007	0.0003	0.0056
**S1_PBR_031**	Failure	0.0111	0.0103	ND	0.0342	0.0113	0.1092	ND	0.0003
**S1_PBR_032**	Failure	0.0005	0.0933	ND	0.0006	0.0093	0.0005	0.0002	0.0077

**S1_PBR_033**	Failure	0.0049	0.0004	ND	0.0135	0.0005	0.0028	0.0122	ND
**S1_PBR_034**	Failure	0.0008	0.0595	ND	0.0005	0.0023	0.0003	0.0007	0.0020
**S1_PBR_035**	Failure	0.0002	0.0265	ND	0.0001	0.0009	0.0014	0.0013	0.0033
**S1_PBR_036**	Failure	0.0117	0.0984	ND	0.0034	0.0034	0.0005	0.0004	0.0033
**S1_PBR_037**	Failure	0.0016	0.0925	ND	0.0007	0.0024	0.0007	0.0004	0.0035
**S1_PBR_038**	Failure	0.0004	0.7538	ND	0.0076	0.0010	0.0002	0.0003	0.0008
**S1_PBR_039**	Failure	0.0018	0.0025	ND	0.0040	0.0007	0.0003	0.0013	0.0003
**S1_PBR_040**	Failure	0.0006	0.0957	ND	0.0012	0.0061	0.0002	0.0005	0.0025
**S1_PBR_041**	Failure	0.0002	0.2640	ND	0.0035	0.0163	0.0007	0.0062	0.0014
**S1_PBR_042**	Failure	0.0001	0.0084	ND	0.0053	0.0009	0.0088	0.0155	0.0003
**S1_PBR_044**	Failure	0.0004	0.1019	ND	0.0003	0.0036	0.0002	0.0001	0.0045
**S1_PBR_045**	Failure	0.0005	0.0746	ND	0.0004	0.0050	0.0007	0.0012	0.0045
**S1_PBR_046**	Failure	0.0002	0.0715	ND	0.0003	0.0046	ND	0.0001	0.0030
**S3_IBR_093**	Stable	ND	0.0001	ND	0.0094	ND	ND	0.0021	0.0005
**S3_IBR_094**	Stable	0.0001	ND	ND	0.0003	ND	ND	ND	ND
**S3_IBR_095**	Stable	0.0002	0.0003	ND	0.0003	0.0018	0.0040	0.0005	0.1892
**S3_IBR_096**	Stable	0.0001	0.0003	ND	0.0006	ND	0.0004	0.0005	0.0006
**S3_IBR_097**	Stable	0.0008	0.0006	ND	0.0024	0.0001	0.0005	0.0023	0.0061
**S3_IBR_098**	Stable	0.0005	0.0007	ND	0.0025	0.0015	0.0026	0.0014	0.0482
**S3_IBR_099**	Stable	0.0008	0.0008	ND	0.0038	0.0014	0.0066	0.0027	0.0010
**S3_IBR_100**	Stable	0.0005	0.0003	ND	0.0014	ND	0.0009	0.0008	0.0005
**S3_IBR_101**	Stable	0.0001	0.0002	ND	0.0004	0.0006	0.0072	0.0028	0.0021
**S3_IBR_102**	Stable	ND	0.0001	ND	0.0009	0.0001	0.0040	0.0073	0.0021
**S3_IBR_103**	Stable	0.0008	0.0004	ND	0.0123	ND	0.0073	0.0120	0.0143
**S3_IBR_104**	Stable	0.0182	0.1045	ND	0.0247	0.0013	0.0061	ND	0.0004
**S3_IBR_105**	Stable	0.0019	0.0023	ND	0.0011	0.0057	0.0042	ND	0.0105
**S3_IBR_106**	Stable	0.0002	0.0006	ND	0.0009	0.0033	0.0043	0.0015	0.0040
**S3_IBR_107**	Stable	0.0008	0.0011	ND	0.0048	ND	0.0039	0.0445	0.1045
**S3_IBR_108**	Stable	0.0025	0.0045	ND	0.0097	0.0006	0.0010	0.0048	0.0531
**S3_IBR_109**	Stable	0.0053	0.0023	ND	0.0075	0.0016	0.0346	0.0002	0.0026
**S3_IBR_110**	Stable	ND	ND	ND	0.0001	0.0006	ND	ND	0.0124
**S3_IBR_111**	Stable	0.0006	ND	ND	ND	0.0001	0.0004	ND	0.0775
**S3_IBR_112**	Stable	0.0001	ND	ND	0.0018	ND	0.0025	ND	0.0046
**S3_IBR_113**	Stable	0.0531	0.0200	ND	0.0263	0.0071	0.0011	ND	0.0228

**S3_IBR_114**	Stable	0.0008	0.0014	ND	0.0314	0.0023	0.0041	0.0055	0.0633
**S3_IBR_115**	Stable	0.0002	0.0009	ND	0.0306	0.0026	0.0045	0.0075	0.0746
**S3_IBR_116**	Stable	0.0015	0.0006	ND	0.0065	ND	0.0016	0.0030	0.0036
**S3_IBR_117**	Stable	0.0001	0.0004	ND	0.0160	0.0045	0.0010	0.0049	0.0095
**S3_IBR_118**	Stable	ND	0.0007	ND	ND	ND	ND	ND	ND
**S3_IBR_119**	Stable	0.0001	0.0002	ND	ND	ND	ND	ND	0.0114
**S3_IBR_120**	Stable	0.0001	0.0012	ND	0.0040	0.0003	ND	ND	0.0047
**S3_IBR_121**	Stable	0.0005	0.0037	ND	0.0006	0.0001	ND	ND	0.0027
**S3_IBR_122**	Stable	ND	0.0003	ND	ND	0.0001	0.0002	0.0001	0.0025
**S3_IBR_123**	Stable	ND	0.0185	ND	0.0030	0.0004	0.0007	0.0004	0.0034
**S3_IBR_124**	Stable	ND	0.0001	ND	ND	ND	ND	ND	0.0010
**S3_IBR_125**	Stable	ND	0.0001	ND	0.0008	0.0006	0.0008	0.0015	0.0011
**S3_IBR_126**	Stable	0.0001	0.0004	ND	ND	ND	0.0002	0.0001	0.0005
**S3_IBR_127**	Stable	0.0004	0.0069	ND	ND	ND	0.0022	0.0020	0.0045
**S3_IBR_128**	Stable	0.0008	0.0025	ND	0.0002	0.0001	0.0007	ND	0.0019
**S3_IBR_129**	Stable	ND	0.0001	ND	0.0007	ND	0.0001	ND	0.0015
**S3_IBR_130**	Stable	0.0001	0.0006	ND	0.0023	0.0001	0.0170	0.0024	0.0026
**S3_IBR_131**	Stable	0.0005	0.0005	ND	0.0014	0.0001	0.0001	0.0006	0.0227
**S3_IBR_132**	Stable	ND	0.0010	ND	ND	0.0002	ND	ND	0.0017
**S3_IBR_133**	Stable	0.0001	0.0008	ND	0.0049	ND	0.0017	0.0004	0.0013
**S3_IBR_134**	Stable	ND	0.0005	ND	0.0010	0.0001	0.0015	0.0025	0.0004
**S3_IBR_135**	Stable	0.0013	0.0004	ND	0.0164	0.0005	0.0011	0.0132	0.0032
**S3_IBR_136**	Stable	ND	0.0005	ND	0.0001	0.0001	0.0821	0.0038	0.0004
**S3_IBR_137**	Stable	0.0001	0.0064	ND	0.0004	ND	0.0012	0.0001	0.0085
**S3_IBR_138**	Stable	0.0004	0.0013	ND	0.0006	ND	0.0009	0.0005	0.0012
**S4_4BR_139**	Stable	0.0127	0.0121	ND	0.0086	ND	0.0173	0.0108	0.0017
**S4_4BR_140**	Stable	0.0007	0.0005	ND	0.0002	0.0002	ND	ND	0.0002
**S4_4BR_141**	Stable	0.1181	0.0829	ND	0.0085	0.0324	0.0046	0.0438	0.0019
**S4_4BR_142**	Stable	0.0001	0.0006	ND	0.0001	ND	0.0001	0.0001	0.0046
**S4_4BR_143**	Stable	0.0002	0.0001	ND	0.0005	ND	0.0002	0.0006	0.0085
**S4_4BR_144**	Stable	0.0013	0.0059	ND	0.0010	0.0005	0.0054	0.0005	0.0151
**S4_4BR_145**	Stable	ND	0.0009	ND	ND	0.0034	0.0008	0.0005	0.0019
**S4_4BR_146**	Stable	0.0004	0.0007	ND	0.0006	0.0005	0.0046	0.0042	0.0169
**S4_4BR_147**	Stable	0.0001	0.0056	ND	0.0012	ND	0.0026	0.0339	0.0002

**S4_4BR_148**	Stable	0.0013	0.0060	ND	0.0071	0.0015	0.0022	0.0031	0.0051
**S4_4BR_149**	Stable	0.0004	0.0005	ND	0.0069	0.0004	0.0006	0.0006	0.0007
**S4_4BR_150**	Stable	0.0001	0.0406	ND	0.0009	0.0005	0.0005	ND	0.0026
**S4_4BR_151**	Stable	0.0001	0.0793	ND	0.0064	0.0078	0.0108	ND	0.0017
**S4_4BR_152**	Stable	0.0020	0.0054	ND	0.0052	0.0032	0.0072	0.0254	0.0070
**S4_4BR_153**	Stable	ND	0.0003	ND	ND	0.0001	ND	0.0002	0.0015
**S4_4BR_154**	Stable	0.0004	0.0045	ND	0.0010	0.0002	0.0015	0.0010	0.0200
**S4_4BR_155**	Stable	0.0037	0.1113	ND	0.0050	0.0017	0.0032	0.0001	0.0042
**S4_4BR_156**	Stable	0.0002	0.0008	ND	0.0003	0.0001	ND	0.0001	0.0035
**S4_4BR_157**	Stable	0.0001	0.0235	ND	0.0007	0.0011	0.0007	0.0010	0.0040
**S4_4BR_158**	Stable	0.0002	0.0110	ND	0.0038	0.0001	0.0016	0.0002	0.0042
**S4_4BR_159**	Stable	0.0024	0.0095	ND	0.0013	0.0013	ND	ND	0.0384
**S4_4BR_160**	Stable	0.0004	0.0017	ND	0.0027	0.0001	0.0005	0.0006	0.0082
**S4_4BR_161**	Stable	0.0001	0.1001	ND	0.0069	0.0015	0.0003	0.0006	0.0056
**S4_4BR_162**	Stable	0.0005	0.0038	ND	0.0062	ND	0.0015	0.0113	0.0119
**S4_4BR_163**	Stable	ND	0.0028	ND	0.0035	0.0005	0.0008	0.0004	0.0037
**S4_4BR_164**	Stable	ND	0.0040	ND	0.0017	ND	0.0009	0.0043	ND
**S4_4BR_165**	Stable	0.0002	0.0035	ND	0.0004	0.0021	0.0001	0.0003	0.0017
**S4_4BR_166**	Stable	ND	0.0006	ND	0.0003	0.0001	ND	0.0002	0.0005
**S4_4BR_167**	Stable	0.0002	0.0061	ND	0.0005	0.0004	0.0001	0.0003	0.0010
**S4_4BR_168**	Stable	0.0001	0.0010	ND	ND	0.0005	0.0009	0.0002	0.0008
**S4_4BR_169**	Stable	0.1456	0.0127	ND	0.0237	0.0042	0.0128	0.0001	0.0004
**S4_4BR_170**	Stable	0.0001	0.0005	ND	0.0006	0.0001	0.0002	0.0001	0.0005
**S4_4BR_171**	Stable	0.0149	0.0040	ND	0.0042	0.0070	0.0055	0.0114	0.0002
**S4_4BR_172**	Stable	0.0001	0.0004	ND	0.0001	0.0001	0.0001	0.0005	0.0001
**S4_4BR_173**	Stable	0.0001	0.0065	ND	0.0003	0.0003	0.0001	0.0004	0.0017
**S4_4BR_174**	Stable	0.0001	0.0020	ND	0.0004	0.0001	0.0002	0.0003	0.0004
**S4_4BR_175**	Stable	ND	0.0345	ND	0.0028	ND	0.0037	0.0135	0.0006
**S4_4BR_176**	Stable	0.0001	0.0015	ND	0.0015	0.0005	0.0005	0.0014	0.0008
**S4_4BR_177**	Stable	0.0007	0.0048	ND	0.0025	0.0005	0.0005	0.0022	0.0008
**S4_4BR_178**	Stable	0.0002	0.0025	ND	0.0006	0.0013	0.0001	0.0045	0.0008
**S4_4BR_179**	Stable	0.0052	0.0047	ND	0.0026	0.0006	0.0017	0.0060	0.0025
**S4_4BR_180**	Stable	0.0037	0.0163	ND	0.0045	0.0010	0.0035	0.0043	0.0023
**S4_4BR_181**	Stable	0.0007	0.0167	ND	0.0012	0.0006	0.0004	0.0020	0.0015

**S4_4BR_182**	Stable	ND	0.0082	ND	0.0049	0.0055	0.0261	0.0102	0.0006
**S4_4BR_183**	Stable	ND	0.0015	ND	0.0006	ND	ND	0.0002	0.0005
**S4_4BR_184**	Stable	0.0058	0.0115	ND	0.0008	0.0032	0.0007	ND	0.0001
