# Supplementary material for: Monitoring of Nitrification in Chloraminated Drinking Water Distribution Systems With Microbiome Bioindicators Using Supervised Machine Learning
Source: Front Microbiol. 2020 Sep 16;11:571009. doi: 10.3389/fmicb.2020.571009 (PMC7526508; doi:10.3389/fmicb.2020.571009)
Supplement: Supplementary file 10 [file Data_Sheet_3.PDF]

Table S5. Test set based on genus-level taxonomic bioindicators of *uncategorized* instances (i.e. operational parameters) obtained from full-scale DWDSs studies. Values represent abundance of each taxon as a ratio of all sequences obtained for each individual sample. Legend: ND = not detected; missing value = ?. †Sequence data was reanalyzed using RDP v16 database.

**Sample** (reference)	**Operational scheme**	**Taxonomy groups** (bioindicators)							
		**Taxa A1**	**Taxa A2**	**Taxa A3**	**Taxa A4**	**Taxa A5**	**Taxa B1**	**Taxa B2**	**Taxa B3**
Arkansas and lower Mississippi Rivers (**Holinger et al., 2014**)									
**CO2-1a**	?	ND	ND	?	ND	ND	ND	ND	0.0056
**CO2-1b**	?	ND	ND	?	ND	ND	ND	ND	0.0095
**CO2-2**	?	ND	ND	?	0.0021	ND	0.0031	ND	0.5849
**CO2-3**	?	ND	0.0008	?	0.0030	ND	ND	0.0008	0.0030
**KS2-1a**	?	ND	0.0077	?	ND	ND	ND	0.0346	0.2308
**KS2-1b**	?	ND	0.0018	?	0.0018	ND	ND	0.0140	0.0595
**KS2-2a**	?	ND	0.0052	?	0.0009	ND	0.0061	0.0506	0.5681
**KS2-2b**	?	ND	0.0032	?	0.0011	ND	0.0032	0.0485	0.4335
**KS3-1a**	?	0.0011	0.1171	?	ND	ND	ND	ND	0.7341
**KS3-1b**	?	0.0011	0.0995	?	0.0011	ND	ND	ND	0.5690
**KS3-2a**	?	ND	0.0585	?	ND	ND	ND	ND	0.0338
**KS3-2b**	?	ND	0.0079	?	ND	ND	0.0032	ND	0.0333
**LA3-1**	?	0.0040	ND	?	ND	ND	ND	ND	ND
**LA3-2**	?	0.0054	ND	?	ND	ND	ND	ND	ND
**LA3-3**	?	0.0043	ND	?	ND	ND	ND	ND	ND
**LA3-4a**	?	0.0206	ND	?	ND	0.0029	ND	ND	ND
**LA3-4b**	?	0.0202	ND	?	ND	ND	ND	ND	ND
**LA4-1a**	?	ND	ND	?	ND	ND	ND	ND	0.2624
**LA4-1b**	?	ND	0.0029	?	ND	ND	0.0029	0.0029	0.2151
**LA4-2a**	?	ND	ND	?	ND	ND	ND	ND	0.6081
**LA4-2b**	?	ND	ND	?	ND	ND	0.2048	ND	0.5127
Ohio River Basin† (**Stanish et al., 2016**)									
**M06S1**	?	ND	0.0311	ND	0.0001	0.0458	0.0038	0.0009	0.0769
**M06S2**	?	ND	0.0218	ND	0.0034	0.0256	0.0016	0.0043	0.0469
**M06S3**	?	0.0006	0.0005	ND	ND	ND	0.0582	0.0169	0.4959

**M06S4**	?	ND	0.0015	ND	0.0005	0.0001	0.0002	0.0096	0.0506
**M06S5**	?	ND	0.0011	ND	0.0001	0.0018	0.0001	0.0012	0.1325
**M07S1**	?	ND	0.0009	ND	0.0021	0.0143	0.0018	0.0041	0.0593
**M07S2**	?	ND	0.0002	ND	0.0005	0.0045	0.0616	0.0014	0.2983
**M07S3**	?	ND	0.0002	ND	ND	0.0020	0.0001	0.0028	0.0701
**M07S4**	?	ND	0.0015	ND	0.0011	0.0065	0.0001	0.0125	0.0588

Ann Arbor, MI† (**Pinto et al., 2014**)

**S.1.1.Apr11**	?	ND	0.0110	ND	ND	ND	ND	0.0020	0.0010
**S.1.1.Aug10**	?	0.0010	0.0030	ND	ND	0.0020	ND	ND	ND
**S.1.1.Aug11**	?	ND	0.0040	ND	ND	0.0040	ND	0.0010	ND
**S.1.1.Dec10**	?	ND	0.0050	ND	ND	0.0010	ND	ND	ND
**S.1.1.Jan11**	?	ND	0.0460	ND	ND	0.0020	ND	ND	ND
**S.1.1.Jul11**	?	0.0010	0.0030	ND	ND	0.0060	ND	ND	0.0010
**S.1.1.Jun11**	?	0.0030	ND	ND	ND	0.0020	ND	ND	ND
**S.1.1.May11**	?	ND	0.0050	ND	ND	ND	ND	ND	ND
**S.1.1.Oct10**	?	0.0030	0.0030	ND	ND	0.0030	ND	ND	ND
**S.1.1.Sep10**	?	0.0010	0.0100	ND	ND	0.0060	ND	ND	ND
**S.1.2.Apr11**	?	ND	0.0110	ND	0.0020	ND	ND	ND	ND
**S.1.2.Aug10**	?	ND	ND	ND	ND	0.0030	ND	ND	ND
**S.1.2.Aug11**	?	0.0040	0.0110	ND	ND	0.0090	ND	ND	ND
**S.1.2.Dec10**	?	ND	0.0130	ND	ND	0.0020	ND	ND	0.0010
**S.1.2.Feb11**	?	ND	0.0360	ND	ND	ND	0.0010	ND	0.0030
**S.1.2.Jan11**	?	ND	0.0510	ND	ND	0.0010	ND	ND	ND
**S.1.2.Jul10**	?	ND	0.0060	ND	ND	0.0010	ND	ND	ND
**S.1.2.Jul11**	?	0.0020	0.0010	ND	ND	0.0070	ND	0.0010	ND
**S.1.2.Jun10**	?	0.0040	0.0050	ND	ND	ND	ND	ND	0.0010
**S.1.2.Jun11**	?	ND	0.0040	ND	ND	0.0050	ND	ND	ND
**S.1.2.Mar11**	?	0.0010	0.0160	ND	0.0030	ND	ND	0.0020	0.0060
**S.1.2.May11**	?	ND	0.0020	ND	ND	0.0010	ND	ND	ND
**S.1.2.Nov10**	?	ND	0.0200	ND	ND	0.0010	ND	0.0010	ND
**S.1.2.Oct10**	?	0.0010	0.0050	ND	ND	0.0040	ND	0.0010	ND
**S.1.2.Sep10**	?	0.0030	0.0080	ND	ND	0.0010	ND	ND	0.0010
**S.1.3.Apr11**	?	ND	0.0050	ND	ND	ND	ND	0.0020	ND

**S.1.3.Aug10**	?	ND	0.0890	ND	0.0010	0.0040	ND	ND	0.0020
**S.1.3.Feb11**	?	ND	0.0200	ND	ND	0.0040	ND	0.0050	0.0030
**S.1.3.Jul10**	?	0.0030	0.0020	ND	0.0020	0.0030	ND	0.0010	0.0020
**S.1.3.Jul11**	?	ND	0.0020	ND	ND	0.0160	ND	ND	0.0060
**S.1.3.Jun10**	?	0.0020	0.0010	ND	ND	ND	ND	0.0010	ND
**S.1.3.Jun11**	?	0.0020	ND	ND	ND	0.0040	ND	ND	0.0020
**S.1.3.Mar11**	?	0.0020	0.0170	ND	0.0010	0.0020	ND	0.0020	ND
**S.2.1.Apr11**	?	ND	0.0090	ND	ND	ND	ND	0.0020	ND
**S.2.1.Aug10**	?	ND	0.0010	ND	ND	0.0010	ND	ND	ND
**S.2.1.Aug11**	?	0.0030	ND	ND	ND	0.0050	ND	ND	ND
**S.2.1.Dec10**	?	0.0010	0.0060	ND	ND	ND	ND	ND	0.0010
**S.2.1.Feb11**	?	ND	0.0160	ND	ND	0.0010	ND	0.0010	ND
**S.2.1.Jul10**	?	ND	0.0030	ND	ND	ND	ND	0.0010	ND
**S.2.1.Jul11**	?	0.0020	0.0020	ND	ND	0.0020	ND	ND	ND
**S.2.1.Jun10**	?	0.0030	0.0010	ND	ND	ND	ND	ND	ND
**S.2.1.Mar11**	?	ND	0.0360	ND	0.0020	0.0020	ND	ND	ND
**S.2.1.May11**	?	0.0010	0.0010	ND	ND	ND	ND	ND	0.0020
**S.2.1.Nov10**	?	ND	0.0200	ND	ND	0.0030	ND	ND	0.0040
**S.2.1.Oct10**	?	0.0030	0.0100	ND	ND	0.0010	ND	ND	ND
**S.2.1.Sep10**	?	ND	0.0060	ND	ND	0.0040	ND	ND	ND
**S.2.2.Apr11**	?	ND	0.0050	ND	ND	ND	ND	ND	ND
**S.2.2.Aug10**	?	0.0020	0.0090	ND	ND	0.0010	ND	ND	ND
**S.2.2.Aug11**	?	0.0010	0.0010	ND	ND	0.0040	ND	ND	ND
**S.2.2.Dec10**	?	ND	0.0080	ND	ND	0.0040	ND	ND	ND
**S.2.2.Feb11**	?	0.0020	0.0210	ND	ND	ND	ND	ND	0.0010
**S.2.2.Jan11**	?	ND	0.0330	ND	ND	0.0030	ND	0.0020	ND
**S.2.2.Jul10**	?	0.0060	0.0080	ND	ND	0.0020	ND	ND	ND
**S.2.2.Jul11**	?	0.0040	ND	ND	ND	0.0020	ND	ND	ND
**S.2.2.Jun10**	?	ND	0.0070	ND	ND	ND	ND	ND	ND
**S.2.2.Jun11**	?	0.0010	ND	ND	ND	0.0020	ND	ND	ND
**S.2.2.Mar11**	?	0.0010	0.0300	ND	0.0010	0.0020	ND	ND	0.0010
**S.2.2.May11**	?	ND	0.0020	ND	ND	ND	ND	ND	ND
**S.2.2.Nov10**	?	0.0020	0.0130	ND	0.0010	0.0020	ND	ND	ND
**S.2.2.Oct10**	?	0.0020	0.0050	ND	0.0010	0.0010	ND	0.0010	0.0010

**S.2.2.Sep10**	?	0.0020	ND	ND	ND	0.0030	ND	0.0010	0.0020
**S.2.3.Apr11**	?	ND	0.0050	ND	ND	ND	ND	ND	0.0020
**S.2.3.Aug10**	?	ND	ND	ND	ND	ND	ND	ND	0.0050
**S.2.3.Aug11**	?	ND	0.0020	ND	ND	0.0020	ND	0.0010	0.0020
**S.2.3.Dec10**	?	ND	0.0040	ND	ND	0.0010	0.0010	ND	0.0040
**S.2.3.Feb11**	?	ND	0.0210	ND	ND	0.0010	ND	0.0010	0.0020
**S.2.3.Jan11**	?	ND	0.0280	ND	ND	ND	ND	ND	ND
**S.2.3.Jul10**	?	0.0010	ND	ND	ND	0.0020	ND	ND	0.0010
**S.2.3.Jul11**	?	0.0010	0.0010	ND	ND	0.0080	ND	0.0020	0.0080
**S.2.3.Jun10**	?	ND	0.0010	ND	ND	ND	ND	ND	ND
**S.2.3.Jun11**	?	ND	0.0020	ND	ND	0.0020	ND	ND	0.0010
**S.2.3.May11**	?	0.0010	0.0050	ND	ND	ND	ND	ND	ND
**S.2.3.Oct10**	?	0.0030	0.0050	ND	ND	0.0160	ND	0.0010	ND
**S.2.3.Sep10**	?	0.0030	0.0090	ND	ND	0.0010	ND	ND	ND
**S.3.1.Apr11**	?	ND	0.0040	ND	ND	ND	ND	ND	0.0020
**S.3.1.Aug10**	?	0.0020	0.0100	ND	0.0010	0.0020	ND	ND	0.0010
**S.3.1.Aug11**	?	ND	0.0760	ND	ND	ND	ND	ND	0.0010
**S.3.1.Dec10**	?	ND	0.0050	ND	ND	0.0020	ND	0.0010	ND
**S.3.1.Feb11**	?	ND	0.0280	ND	ND	0.0020	ND	ND	0.0010
**S.3.1.Jul10**	?	ND	0.0020	ND	ND	0.0010	ND	0.0010	0.0010
**S.3.1.Jun10**	?	0.0030	0.0070	ND	ND	0.0050	ND	ND	ND
**S.3.1.Mar11**	?	ND	0.0280	ND	ND	0.0020	ND	ND	0.0010
**S.3.1.May11**	?	ND	ND	ND	ND	ND	ND	ND	0.0010
**S.3.1.Nov10**	?	ND	0.0160	ND	ND	0.0040	ND	0.0010	0.0080
**S.3.1.Oct10**	?	0.0030	0.0520	ND	ND	0.0060	ND	0.0010	0.0030
**S.3.1.Sep10**	?	0.0020	0.0170	ND	ND	0.0030	ND	ND	0.0010
**S.3.2.Apr11**	?	0.0010	0.0050	ND	ND	0.0010	ND	ND	ND
**S.3.2.Aug10**	?	0.0030	ND	ND	ND	0.0020	ND	0.0010	ND
**S.3.2.Aug11**	?	ND	0.0040	ND	ND	0.0060	ND	ND	0.0010
**S.3.2.Dec10**	?	ND	0.0120	ND	ND	0.0010	ND	ND	0.0040
**S.3.2.Feb11**	?	0.0020	0.0330	ND	ND	0.0010	ND	ND	0.0060
**S.3.2.Jan11**	?	ND	0.0610	ND	0.0010	0.0010	ND	ND	ND
**S.3.2.Jul10**	?	ND	ND	ND	ND	ND	ND	ND	ND
**S.3.2.Jul11**	?	0.0010	ND	ND	ND	0.0030	ND	ND	0.0020

**S.3.2.Jun10**	?	ND	0.0010	ND	0.0030	ND	ND	ND	ND
**S.3.2.Jun11**	?	0.0040	ND	ND	ND	0.0020	ND	ND	ND
**S.3.2.Mar11**	?	0.0020	0.0380	ND	ND	ND	ND	ND	0.0010
**S.3.2.May11**	?	ND	0.0010	ND	ND	ND	ND	ND	ND
**S.3.2.Nov10**	?	0.0020	0.0040	ND	ND	0.0040	ND	0.0010	ND
**S.3.2.Oct10**	?	0.0030	0.0090	ND	ND	0.0060	ND	ND	ND
**S.3.2.Sep10**	?	0.0010	0.0120	ND	ND	0.0010	ND	0.0020	ND
**S.3.3.Apr11**	?	ND	0.0130	ND	ND	ND	ND	ND	0.0020
**S.3.3.Aug10**	?	0.0010	0.0040	ND	ND	0.0030	ND	ND	ND
**S.3.3.Aug11**	?	0.0030	0.0020	ND	ND	0.0050	ND	0.0010	ND
**S.3.3.Dec10**	?	ND	0.0020	ND	ND	ND	ND	0.0030	ND
**S.3.3.Feb11**	?	ND	0.0200	ND	ND	0.0020	ND	0.0060	0.0030
**S.3.3.Jan11**	?	ND	0.0300	ND	ND	ND	ND	ND	ND
**S.3.3.Jul10**	?	0.0030	0.0040	ND	ND	0.0010	ND	ND	ND
**S.3.3.Jul11**	?	ND	ND	ND	ND	0.0030	ND	ND	0.0010
**S.3.3.Jun10**	?	ND	ND	ND	ND	ND	ND	ND	0.0010
**S.3.3.Jun11**	?	ND	ND	ND	ND	0.0040	ND	ND	ND
**S.3.3.Mar11**	?	ND	0.0300	ND	ND	ND	ND	ND	ND
**S.3.3.May11**	?	0.0010	ND	ND	ND	0.0010	ND	0.0010	ND
**S.3.3.Nov10**	?	ND	0.0180	ND	ND	0.0080	0.0010	ND	ND
**S.3.3.Oct10**	?	ND	0.2430	ND	ND	0.0070	ND	0.0010	0.0020
**S.3.3.Sep10**	?	0.0020	0.0020	ND	ND	0.0030	ND	0.0010	ND

Eastern USA† (**Ji et al., 2015**)

**E.B.1.1D**	?	0.0009	0.0149	ND	0.1112	0.0020	0.0285	0.0268	0.1383
**E.B.1.2D**	?	0.0017	0.0149	ND	0.1284	0.0026	0.0288	0.0182	0.1246
**E.B.1.3D**	?	0.0009	0.0157	ND	0.1455	0.0032	0.0294	0.0205	0.1101
**E.B.2.1D**	?	0.0154	0.0273	ND	0.1880	0.0029	0.0571	0.0227	0.0973
**E.B.2.2D**	?	0.0058	0.0188	ND	0.2368	0.0023	0.0543	0.0191	0.1051
**E.B.2.3D**	?	0.0064	0.0145	ND	0.2180	0.0036	0.0633	0.0228	0.1059
**E.B.3.1D**	?	0.0015	0.0218	ND	0.2035	0.0022	0.0610	0.0355	0.3084
**E.B.3.2D**	?	0.0012	0.0137	ND	0.2286	0.0029	0.0638	0.0294	0.2762
**E.B.3.3D**	?	0.0017	0.0148	ND	0.2494	0.0037	0.0534	0.0312	0.2085
**E.CS.1.1D**	?	0.0005	0.0069	ND	0.1786	0.0006	0.0334	0.0833	0.0964

**E.CS.1.2D**	?	0.0008	0.0100	ND	0.2344	0.0008	0.0375	0.0563	0.1112
**E.CS.1.3D**	?	0.0023	0.0097	ND	0.1911	0.0015	0.0236	0.0646	0.1274
**E.CS.2.1D**	?	0.0005	0.0022	ND	0.1432	0.0014	0.0168	0.1204	0.1113
**E.CS.2.2D**	?	0.0010	0.0043	ND	0.1317	0.0012	0.0120	0.1336	0.1181
**E.CS.2.3D**	?	0.0003	0.0034	ND	0.1519	0.0014	0.0193	0.1733	0.0906
**E.CS.3.1D**	?	0.0008	0.0103	ND	0.1952	0.0024	0.0220	0.0431	0.0989
**E.CS.3.2D**	?	0.0010	0.0145	ND	0.2313	0.0014	0.0255	0.0434	0.0565
**E.CS.3.3D**	?	0.0014	0.0056	ND	0.2072	0.0014	0.0118	0.0727	0.0594
**E.I.1D**	?	0.0168	0.0020	ND	0.0211	0.0040	0.0314	0.0152	0.0062
**E.I.2D**	?	0.0146	0.0103	ND	0.0143	0.0031	0.0214	0.0150	0.0063
**E.I.3D**	?	0.0058	0.0202	ND	0.0235	0.0042	0.0223	0.0140	0.0028
**E.LC.1.1D**	?	0.0009	0.0207	ND	0.2492	0.0021	0.0504	0.0237	0.2714
**E.LC.1.2D**	?	0.0014	0.0058	ND	0.2666	0.0024	0.0675	0.0179	0.2071
**E.LC.1.3D**	?	0.0014	0.0100	ND	0.3103	0.0019	0.0614	0.0180	0.2122
**E.LC.2.1D**	?	0.0009	0.0166	ND	0.1747	0.0021	0.0343	0.0141	0.2530
**E.LC.2.2D**	?	0.0032	0.0117	ND	0.1615	0.0047	0.0287	0.0174	0.2391
**E.LC.2.3D**	?	0.0018	0.0062	ND	0.1845	0.0020	0.0308	0.0394	0.1521
**E.LC.3.1D**	?	0.0006	0.0079	ND	0.1238	0.0016	0.0342	0.0054	0.1555
**E.LC.3.2D**	?	0.0031	0.0066	ND	0.2324	0.0025	0.0616	0.0113	0.1784
**E.LC.3.3D**	?	ND	0.0074	ND	0.0436	0.0010	0.0821	0.0212	0.2516

New Orleans, LA[†] (**Hull et al., 2017**)

**A_NO26**	?	ND	0.0006	ND	0.0001	ND	ND	ND	0.0016
**A_NO27**	?	ND	0.0007	ND	ND	ND	0.0006	0.0005	0.0021
**A_NO28**	?	ND	0.0004	0.0001	ND	ND	ND	ND	0.0142
**A_NO29**	?	ND	0.0002	ND	0.0001	ND	ND	0.0002	0.0195
**A_NO31**	?	0.0001	0.0001	ND	0.0039	ND	ND	0.0013	0.0049
**C_NO16**	?	0.0001	0.0001	0.0002	ND	0.0002	ND	0.0051	0.0091
**C_NO17**	?	ND	ND	ND	ND	0.0005	ND	ND	0.0107
**C_NO18**	?	ND	ND	0.0001	0.0002	ND	ND	ND	0.0009
**C_NO19**	?	ND	ND	ND	ND	ND	ND	0.0002	0.1259
**C_NO20**	?	ND	ND	0.0001	ND	0.0002	ND	0.0004	0.0036
**C_NO21**	?	ND	ND	ND	ND	ND	ND	0.0013	0.4707
**C_NO23**	?	ND	ND	ND	ND	ND	ND	0.0001	0.0016

**C_NO24**	?	ND	0.0001	ND	ND	ND	ND	0.0001	0.0022
**C_NO25**	?	ND	0.0004	ND	ND	0.0002	ND	0.0067	0.1191
**C_NO32**	?	ND	ND	ND	ND	ND	ND	0.0005	0.0541
**C_NO33**	?	ND	0.0001	ND	ND	0.0002	0.0002	0.0001	0.0382
**C_NO34**	?	0.0001	ND	ND	ND	0.0001	0.0001	0.0008	0.0116
**C_NO35**	?	ND	0.0002	ND	0.0002	0.0001	ND	0.0012	0.0060
**C_NO36**	?	ND	0.0001	ND	ND	ND	ND	0.0001	0.0595
**C_NO37**	?	ND	ND	0.0001	ND	0.0001	0.0001	0.0002	0.0034
**C_NO38**	?	ND	ND	0.0001	0.0005	0.0001	ND	0.0018	0.0244
**C_NO39**	?	ND	ND	ND	ND	0.0011	ND	0.0194	0.4369
**C_NO40**	?	ND	0.0001	0.0002	ND	0.0002	0.0001	ND	0.1095
**C_NO41**	?	ND	0.0001	ND	ND	0.0001	ND	ND	0.0024
**C_NO42**	?	ND	0.0001	ND	0.0002	0.0008	ND	0.0012	0.0074
**C_NO43**	?	ND	ND	ND	0.0001	ND	ND	ND	0.0380
**C_NO44**	?	ND	ND	0.0001	ND	ND	0.0001	0.0002	0.0033
**C_NO45**	?	ND	0.0001	0.0001	ND	0.0001	ND	0.0001	0.0276
**C_NO46**	?	ND	ND	0.0001	ND	0.0001	0.0001	0.0004	0.0566
**C_NO47**	?	ND	ND	ND	ND	0.0002	ND	0.0002	0.0122
**C_NO48**	?	ND	0.0004	ND	ND	ND	ND	ND	0.0045
**C_NO49**	?	ND	0.0001	0.0001	0.0002	0.0001	ND	0.0002	0.0196
**C_NO50**	?	ND	0.0001	ND	ND	0.0001	ND	ND	0.1174
**C_NO51**	?	ND	ND	ND	ND	0.0002	ND	0.0001	0.0493
**C_NO52**	?	0.0042	0.0041	ND	0.0002	0.0051	ND	0.0006	0.0014
**C_NO53**	?	ND	ND	ND	ND	0.0002	ND	0.0001	0.3029
**C_NO54**	?	ND	ND	ND	ND	0.0004	0.0001	0.0012	0.2919
**C_NO55**	?	ND	0.0002	ND	ND	ND	ND	ND	0.0015
**C_NO56**	?	ND	ND	0.0001	0.0008	0.0005	0.0001	0.0001	0.0047
**C_NO57**	?	ND	0.0001	ND	0.0001	ND	ND	0.0001	0.0061

Urbana, IL[†] (**Hwang et al., 2012**)

**S10_C0**	?	ND	ND	ND	0.0007	ND	ND	ND	0.0007
**S10_N1**	?	ND	ND	ND	ND	ND	ND	ND	ND
**S10_N2**	?	ND	ND	ND	ND	ND	ND	ND	ND
**S10_S1**	?	ND	ND	ND	0.0020	ND	ND	ND	0.0013

**S10_S2**	?	ND	ND	ND	0.0040	ND	ND	0.0007	0.0007
**S11_C0**	?	ND	ND	ND	ND	ND	ND	ND	ND
**S11_N1**	?	ND	ND	ND	ND	ND	ND	ND	ND
**S11_N2**	?	ND	ND	ND	0.0073	ND	ND	ND	0.0013
**S11_S1**	?	ND	ND	ND	ND	ND	ND	ND	ND
**W10_C0**	?	ND	ND	ND	0.0087	0.0013	ND	0.0007	0.0007
**W10_N1**	?	ND	ND	ND	0.0107	0.0020	ND	ND	ND
**W10_N2**	?	ND	ND	ND	0.0060	0.0027	ND	ND	ND
**W10_S1**	?	ND	ND	ND	0.0020	ND	ND	ND	ND
**W10_S2**	?	ND	ND	ND	0.0013	ND	ND	ND	ND
