# Supplementary material for: Monitoring of Nitrification in Chloraminated Drinking Water Distribution Systems With Microbiome Bioindicators Using Supervised Machine Learning
Source: Front Microbiol. 2020 Sep 16;11:571009. doi: 10.3389/fmicb.2020.571009 (PMC7526508; doi:10.3389/fmicb.2020.571009)
Supplement: Supplementary file 11 [file Data_Sheet_4.PDF]

# MONITORING OF NITRIFICATION IN CHLORAMINATED DRINKING WATER DISTRIBUTION SYSTEMS WITH MICROBIOME BIOINDICATORS USING SUPERVISED MACHINE LEARNING

V. Gomez-Alvarez and R. P. Revetta

U.S. Environmental Protection Agency, Office of Research and Development, Cincinnati, Ohio 45268

## SUPPLEMENTAL MATERIAL

1) SUPPLEMENTARY MATERIALS and METHODS

2) SUPPLEMENTARY TABLES

**Table S1.** Water quality summary.

**Table S2.** Relative abundances of genus-level assigned bioindicators.

**Table S3.** Training set with relative abundances of genus-level assigned bioindicators.

**Table S4.** Test set of *categorized* instances with relative abundances of genus-level assigned bioindicators.

**Table S5.** Test set of *uncategorized* instances with relative abundances of genus-level assigned bioindicators.

3) SUPPLEMENTARY FIGURES

**Figure S1.** Schematic of the WEKA Knowledge Flow Interface.

**Figure S2.** Relative abundance of differentially abundant OTU-level assigned bioindicators.

**Figure S3**. Rarefaction curves for the DWDS bulk water microbiomes.

**Figure S4.** LEfSe, ROC and PRC analyses of bioindicators based on genus-level taxonomic groups.

**Figure S5.** ROC and PRC analyses of bioindicators based on genus-level taxonomic groups using publicly available reference databases.

## SUPPLEMENTARY MATERIALS and METHODS

### Drinking water distribution system (DWDS) simulator

A semi-closed pipe-loop DWDS simulator (**Gomez-Alvarez et al., 2016**) was operated through successive operational schemes (see schematic). Pipe-loop feed water was created from a free chlorine municipal drinking water (DW) source. The DW source was amended as necessary with ammonium sulfate and sodium hypochlorite to generate a target 3 mg $L^{-1}$ monochloramine (Stable [SS; SI and SII] and Failure [SF]) or free chlorine (Restore [SR]) residual concentration. For monochloramine formation, a 4.5:1 chlorine to ammonia-nitrogen mass ratio ($Cl_2$:N) was used. The operational scheme sequence was designed to parallel a practical scenario where a chloraminated drinking water system progresses from normal operation where a chloramine residual is maintained in the pipe-loop (SS) to a failure period where no chloramine residual is maintained as a result of nitrification (SF). Subsequently, the drinking water system operation is modified by switching disinfectants from chloramine to free chlorine to eliminate nitrification and maintain a disinfectant residual (SR). After a period, the drinking water utility then switches back to chloramine and resumes normal operation (SS). To accelerate the development of nitrification and disinfectant residual loss, the temperature of the pipe-loop water was increased (from 18°C to 24°C) for the Failure operational scheme.

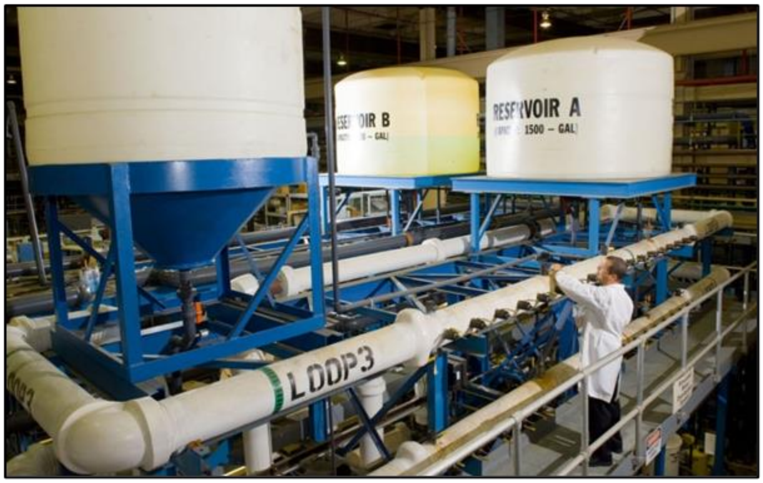


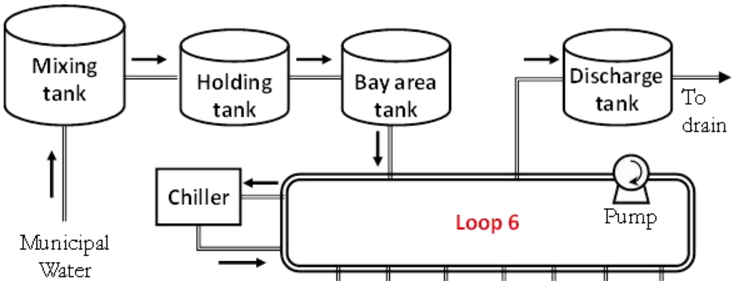


### Water quality

Bulk water (BW) concentrations were analyzed from a composite of two samples taken from two different ports in the pipe-loop. Temperature, pH, turbidity, and ORP measurements were taken from online sensors. Nitrate and phosphate analyses were performed with a discrete colorimetric SmartChem 200 autoanalyzer (Westco Scientific, Danbury CT) using the EPA methods 353.2 (**USEPA 1993a**) and 365.1 (**USEPA 1993b**). Chemical concentrations for free chlorine (HACH Method 8021), monochloramine and free ammonia (HACH Method 10200), total chlorine

(HACH Method 8167), and nitrite (HACH Method 8507) were determined with a HACH DR/2400 Portable Spectrophotometer (HACH, Loveland, CO, USA).

**Sample Collection**

Duplicate BW samples (3 L) were collected from two ports in the pipe-loop DWDS simulator using sterile polypropylene bottles (Nalgene, Rochester, NY). Samples were individually filtered on-site through polycarbonate membranes (47 mm diameter, 0.22 µm pore size). Membranes were individually overlaid with 100 µmol $L^{-1}$ Propidium Monoazide (PMA) and incubated in the dark for 20 min, followed by a 15 min exposure to 460 nm light (**Hellein et al., 2012**). PMA can inhibit PCR amplification of DNA from membrane-compromised cells and effectively discriminates between live and dead bacteria (**Nocker et al., 2010**). Membranes were stored and transported on ice for DNA processing.

**DNA Extraction and Sequencing**

Total DNA was extracted using the MoBio PowerWater® DNA Isolation Kit. DNA extractions followed the manufacturer's instructions (MoBio Laboratories, Solana Beach, CA). DNA concentrations were measured using the Qubit® Fluorometer (Life Technologies, Carlsbad, CA) and stored at -80°C. The V4 region of the 16S rRNA sequence was amplified using the bacterial primer set 515F and 806R (**Caporaso et al., 2012**). Paired-end 125 bp libraries were prepared using the Illumina MiSeq® Reagent v2 (500-cycles) kit on the MiSeq platform (Illumina Inc., San Diego, CA).

**16S rRNA sequence analysis**

Reads were analyzed using the software MOTHUR v1.37.6 (**Schloss et al., 2009**) and were screened following the procedure described in **Gomez-Alvarez et al. (2016)**. Briefly, fastq files with forward and reverse reads were used to form contigs. Reads were screened and removed if they (i) had a length less than 292 bp, (ii) contained ambiguous bases (N's), (iii) contained homopolymers greater than 7 bases, (iv) were identified as chimera, or (v) were classified as unknown, Chloroplasts, or Mitochondria. Reads were aligned against the SILVA SEED release 123 reference dataset and grouped with 97% sequence identity as the cut-off point for each Operational Taxonomic Unit (OTU). Taxonomic classification was obtained using the Ribosomal Database Project (RDP v16) reference database. The sequences and taxonomic outlines for the RDP hierarchies were downloaded from the MOTHUR website (https://www.mothur.org). Prior to community analysis, samples were rarefied to the smallest dataset (5 000 reads).

**Biomass quantification**

Total ATP of bacterial cells was used as a surrogate for biomass present in BW samples. The amount of ATP was determined by ATP-bioluminescence quantification using the Promega BacTiter-Glo™ Microbial Cell Viability Assay kit (Promega, Madison, WI), following the protocol by **Berney et al. (2008)**. Briefly, the BacTiter-Glo™ Buffer was mixed with the lyophilized BacTiter-Glo™ Substrate and stored over night at room temperature. 100 mL of bulk water (BW) sample and an equal volume of BacTiter-Glo™ reagent (stored on ice) were warmed separately for 2 min in a 30°C water bath. BW and reagent were mixed, and the luminescence of the sample was immediately measured with a GloMax® 96 Microplate Luminometer (Promega, Madison, WI). All samples were analyzed in triplicate and free ATP (in BW) was also measured. A calibration curve was prepared with dilutions of pure ATP (Promega, Madison, WI).

## REFERENCES


Berney, M., Vital, M, Hülshoff, I., Weilenmann, H. U., Egli, T., Hammes, F. (2008). Rapid, cultivation-independent assessment of microbial viability in drinking water. *Water Res.* 42, 4010-4018.

Caporaso, J. G., Lauber, C. L., Walters, W. A., Berg-Lyons, D., Huntley, J., Fierer, N., Owens, S. M., Betley, J., Fraser, L., Bauer, M., Gormley, N., Gilbert, J. A., Smith, G., Knight, R. (2012). Ultra-high-throughput microbial community analysis on the Illumina HiSeq and MiSeq platforms. *ISME J.* 6, 1621-1624.

Gomez-Alvarez, V., Pfaller, S., Pressman, J. G., Wahman, D. G., Revetta, R. P. (2016). Resilience of microbial communities in a simulated drinking water distribution system subjected to disturbances: role of conditionally rare taxa and potential implications for antibiotic-resistant bacteria. *Environ. Sci.: Water Res. Technol.* 2, 645-657.

Hellein, K. N., Kennedym E. M., Harwood, V. J., Gordon, K. V., Wang, S. Y., Lepo, J. E. (2012). A filter-based propidium monoazide technique to distinguish live from membrane-compromised microorganisms using quantitative PCR. *J. Microbiol. Meth.* 89 ,76-78.

Holinger, E. P., Ross, K. A., Robertson, C. E., Stevens, M. J., Harris, J. K., Pace, N. R. (2014). Molecular analysis of point-of-use municipal drinking water microbiology. *Water Res.* 49, 225-235.

Hull, N. M., Holinger, E. P., Ross, K. A., Robertson, C. E., Harris, J. K., Stevens, M. J., Pace, N. R. (2017). Longitudinal and source-to-tap New Orleans, LA, U.S.A. drinking water microbiology. *Environ. Sci. Technol.* 51, 4220-4229.

Hwang, C., Ling, F., Andersen, G. L., LeChevallier, M. W., Liu, W. T. (2012). Microbial community dynamics of an urban drinking water distribution system subjected to phases of chloramination and chlorination treatments. *Appl. Environ. Microbiol.* 78, 7856-7865.

Ji, P., Parks, J., Edwards, M. A., Pruden, A. (2015). Impact of water chemistry, pipe material and stagnation on the building plumbing microbiome. *PLoS One* 10, e0141087.

Nocker, A., Richter-Heitmann, T., Montijn, R., Schuren, F., Kort, R. (2010). Discrimination between live and dead cells in bacterial communities from environmental water samples analyzed by 454 pyrosequencing. *Int. Microbiol.* 13 59-65.

Pinto, A. J., Schroeder, J., Lunn, M., Sloan, W., Raskin, L. (2014). Spatial-temporal survey and occupancy-abundance modeling to predict bacterial community dynamics in the drinking water microbiome. *MBio* 5, e01135-14.

Schloss, P. D., Westcott, S. L., Ryabin, T., Hall, J. R., Hartmann, M., Hollister, E. B., Lesniewski, R. A., Oakley, B. B., Parks, D. H., Robinson, C. J., Sahl, J. W., Stres, B., Thallinger, G. G., Van Horn, D. J., Weber, C. F. (2009). Introducing mothur: open-source, platform-independent, community-supported software for describing and comparing microbial communities. *Appl. Environ. Microbiol.* 75, 7537-7541.

Shaw, J. L., Monis, P., Weyrich, L. S., Sawade, E., Drikas, M., Cooper, A. J. (2015). Using amplicon sequencing to characterize and monitor bacterial diversity in drinking water distribution systems. *Appl. Environ. Microbiol.* 81, 6463-6473.

Stanish, L. F., Hull, N. M., Robertson, C. E., Harris, J. K., Stevens, M. J., Spear, J. R., Pace, N. R. (2016). Factors influencing bacterial diversity and community composition in municipal drinking waters in the Ohio River basin, USA. *PLoS One* 11, e0157966.

USEPA (United States Environmental Protection Agency). (1993a). Method 353.2, Revision 2.0: Determination of Nitrate-Nitrite by Automated Colorimetry, ed. J. W. O'Dell. Cincinnati, OH: Office of Research and Development.

USEPA (United States Environmental Protection Agency). (1993b). Method 365.1: Determination of Phosphorus by Semi-Automated Colorimetry, ed. J. W. O'Dell. Cincinnati, OH: Office of Research and Development.

Wang, H., Proctor, C. R., Edwards, M. A., Pryor, M., Santo Domingo, J. W., Ryu, H., Camper, A. K., Olson, A., Pruden, A. (2014). Microbial community response to chlorine conversion in a chloraminated drinking water distribution system. *Environ Sci. Technol.* 48, 10624-10633.
